# Distinct roles of Argonaute in the green alga *Chlamydomonas* reveal evolutionary conserved mode of miRNA-mediated gene expression

**DOI:** 10.1038/s41598-019-47415-x

**Published:** 2019-07-31

**Authors:** Betty Y.-W. Chung, Adrian Valli, Michael J. Deery, Francisco J. Navarro, Katherine Brown, Silvia Hnatova, Julie Howard, Attila Molnar, David C. Baulcombe

**Affiliations:** 1 Department of Plant Sciences, University of Cambridge, Cambridge, CB2 3EA United Kingdom; 2 Present Address: Department of Pathology, University of Cambridge, Cambridge, CB2 1QP United Kingdom; 3 Present Address: Department of Plant Molecular Genetics, Spanish National Centre for Biotechnology, Madrid, 28049 Spain; 4 Cambridge System Biology Centre and Department of Biochemistry, University of Cambridge, Cambridge, CB2 1GA United Kingdom; 5 Institute of Molecular Plant Sciences, University of Edinburgh, Edinburgh, EH9 3BF United Kingdom

**Keywords:** Evolution, miRNAs

## Abstract

The unicellular green alga *Chlamydomonas reinhardtii* is evolutionarily divergent from higher plants, but has a fully functional silencing machinery including microRNA (miRNA)-mediated translation repression and mRNA turnover. However, distinct from the metazoan machinery, repression of gene expression is primarily associated with target sites within coding sequences instead of 3′UTRs. This feature indicates that the miRNA-Argonaute (AGO) machinery is ancient and the primary function is for post transcriptional gene repression and intermediate between the mechanisms in the rest of the plant and animal kingdoms. Here, we characterize AGO2 and 3 in *Chlamydomonas*, and show that cytoplasmically enriched Cr-AGO3 is responsible for endogenous miRNA-mediated gene repression. Under steady state, mid-log phase conditions, Cr-AGO3 binds predominantly miR-C89, which we previously identified as the predominant miRNA with effects on both translation repression and mRNA turnover. In contrast, the paralogue Cr-AGO2 is nuclear enriched and exclusively binds to 21-nt siRNAs. Further analysis of the highly similar Cr-AGO2 and Cr-AGO 3 sequences (90% amino acid identity) revealed a glycine-arginine rich N-terminal extension of ~100 amino acids that, given previous work on unicellular protists, may associate AGO with the translation machinery. Phylogenetic analysis revealed that this glycine-arginine rich N-terminal extension is present outside the animal kingdom and is highly conserved, consistent with our previous proposal that miRNA-mediated CDS-targeting operates in this green alga.

## Introduction

The diverse natural roles for RNA-silencing in eukaryotes range from defense against viruses to the regulation of gene expression and chromatin remodeling^1–9^. The central player of this pathway is the evolutionarily conserved ribonuclease protein – Argonaute (AGO). Sequence specificity of the RNA-silencing pathway is facilitated by 20–30 nucleotide (nt) small RNAs (sRNAs) that bind to the AGO protein and guide it to RNAs with complementary target sites, leading to silencing of the target messengers via translational repression and/or degradation.

AGO proteins are present almost throughout the eukaryote kingdom, thus suggesting that the RNA silencing pathway is ancient and was present in the last eukaryotic common ancestor, where it most likely functioned in defense against viruses and transposons^10^. AGO proteins are also present in prokaryotes but are associated with DNA instead of RNA^11,12^. Eukaryote AGO proteins are part of the PIWI-protein superfamily, defined by the presence of a PIWI (P element-induced wimpy testes) domain^12^. They have been divided into four major clades – the Trypanosoma AGO clade, the WAGO (worm Argonaute) clade, the AGO-like clade and the PIWI-like clade^12^. AGO-like proteins are present in plants, fungi and animals, whereas PIWI-like are specific to the germline in animals.

Four domains are conserved in eukaryotic AGOs: the N-domain, which plays a critical role in target cleavage and in the dissociation of strands after cleavage^13–16^; the PAZ domain, which binds to the 3′ end of the associated small RNA^17,18^; the MID domain which contains a basic binding pocket that recognizes the 5′ phosphate of the associated small RNA^19^ and may discriminate between the various 5′ end nucleotides^20,21^; and the PIWI domain, which contains the RNase H-like active site and the catalytic triad DDX (where X is usually aspartic acid or histidine or occasionally lysine)^22,23^.

The haploid unicellular green alga *Chlamydomonas reinhardtii* has a complex set of small RNAs and diverse RNA silencing pathways ranging from miRNA-mediated cleavage of target mRNA to translational repression^24–29^. The *Chlamydomonas* genome contains three *AGO* genes with *Cr-AGO1* being more divergent than *Cr-AGO2* and *3*. AGO1 has a likely role in genome defense against transposable elements^30^, while Cr-AGO3 is probably responsible for miRNA-mediated transcript cleavage and translational repression^31^.

In this work, we further characterize *Chlamydomonas* AGO proteins in order to understand the evolution of this protein family in eukaryotes. We show that Cr-AGO2 is preferentially nuclear localized, while Cr-AGO3 is cytoplasmic enriched and associates with polysomes. IP experiments confirmed that Cr-AGO3 associates with miRNAs, while Cr-AGO2 exclusively binds siRNAs. The most prevalent miRNA bound to Cr-AGO3 is miR-C89, the only known endogenous miRNA that mediates both translation repression and mRNA turnover^1^. We also further characterize the structure of the three AGO proteins in *Chlamydomonas* revealing differences in length of an unstructured extension of the N-domain, enriched in glycine and arginine residues (GR-N). We extend the analysis of this domain to all annotated AGO proteins, revealing that while this domain is generally absent in AGO proteins from archaea, bacteria, microsporidia and animals, it is conserved in almost all other eukaryotes. Based on these findings we propose that the GR-N domain might facilitate coding region targeting by AGO proteins as demonstrated in our previous study^1^.

## Results and Discussion

### Computational analysis of *Chlamydomonas* AGO domain structure

**Figure 1:**
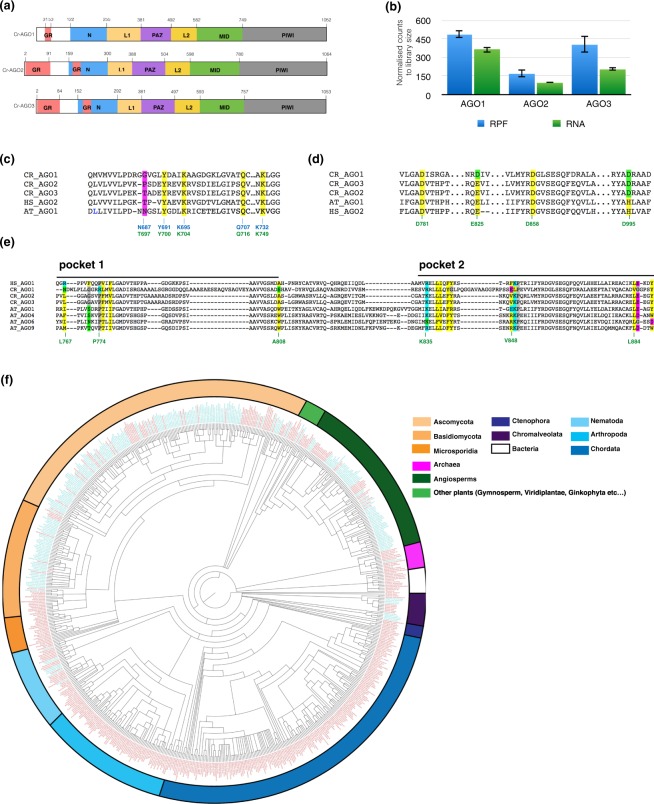
Structural features of *Chlamydomonas* Argonautes: (**a**) Domain structure of AGO proteins in *Chlamydomonas reinhardtii*. Domains N, L1, PAZ, L2, MID and PIWI were identified by threading the amino acid sequence onto the HS-AGO crystal structure (PDB:4W5Q), followed by extraction of the corresponding sequence for each domain. The GR-N domain was determined as described in the methods section. (**b**) Relative expression levels in terms of total translation and RNA abundance (blue and green, respectively) for all Cr-AGOs (biological replicates of wild-type strain CC-1883, data from previous study^1^). (**c**) Sequence alignment showing conserved 5′-phosphate-binding residues of the Argonaute MID domains from *Chlamydomonas*, Human AGO2 and *Arabidopsis* AGO1. Residues Y, K, Q and K required for 5′ phosphate recognition are conserved in all Cr-AGOs. The non-conserved residue involved in 5′ nt recognition, is highlighted in magenta. Coordinates position in blue and green are corresponding coordinates in At-AGO1 and Cr-AGO3, respectively. (**d**) Sequence alignment showing conserved catalytic residues (yellow) of Argonaute PIWI domains across various species. Non-conserved residues aligning to the catalytic sites are highlighted in green. Corresponding Cr-AGO3 coordinates are in green. (**e**) Sequence alignment of tandem tryptophan-pockets in the PIWI domain. Residues forming the binding pockets are highlighted. Hydrophobic, polar, positively charged and negatively charged residues are highlighted in yellow, green, cyan and magenta respectively. All other residues are highlighted in grey. Corresponding Cr-AGO3 coordinates are in green. (**f**) Phylogenic tree of life (source NCBI) with all species containing at least one Argonaute protein with an N-terminal GR-N domain highlighted in blue. Species that do not contain any Argonaute with an N-terminal GR-N domain are highlighted in red.

The *Chlamydomonas reinhardtii* genome encodes three AGOs - Cr-AGO1 (109 kDa), Cr-AGO2 (109 kDa) and Cr-AGO3 (108 kDa) [phytozome V10.1]. Similar to other eukaryotic AGOs, each protein contains all conserved domains (in order of N to C terminus) - N, PAZ, MID and PIWI, where the PAZ domain is linked to the N and MID domains by two linker regions, linker 1 and 2 (L1 and L2), respectively (Fig. 1a, Supplementary Fig. 1a). From the ribosome profiling data in our previous study^1^, which is essentially a direct measurement of protein synthesis, it appears that Cr-AGO1 is the most abundant of the three AGO proteins, followed by Cr-AGO3 then Cr-AGO2, which is in agreement with RNA abundance (Fig. 1b).

In order to characterize Cr-AGOs and identify structural differences that might indicate functional specialization, we estimated Cr-AGO structures by threading the amino acid sequences through the crystalized human AGO2 structure^32^. We focused on three localized signatures that characterize specific AGO functions: (1) the MID-domain residues known to interact with the 5′ phosphate residue for small RNAs and can be responsible for small RNA sorting; (2) the catalytic tetrad in the PIWI domain required for target cleavage; and (3) the tryptophan binding pockets utilised for heterodimer formation with tryptophan-containing proteins.

All three Cr-AGOs possess the highly conserved 5′ phosphate binding residues in the MID domain^33^ - corresponding to K127, Y123, Q159 and K163 in *A*. *fulgidus* AGO^34^ or Y691, K695, Q707 and K732 in *A*. *thaliana* AGO1 which also contributes to its 5′ nt selectivity^20^. This finding suggests that all three Cr-AGOs may interact with small RNAs containing a 5′ phosphate group. The corresponding key residue affecting binding of the 5′ residue of the sRNA (N687 in At-AGO1) differs between Cr-AGO2 and 3 which suggested that, similar to At-AGOs, Cr-AGO2 and 3 have the capacity for differential sRNA binding specificity (Fig. 1c and Supplementary Fig. 1b). Further, analysis of Hs-AGO2 demonstrates that I365 on helix 7 that promotes the targeting rule based on seed-pairing^35^. Both helix 7 and residue I365 are highly conserved in Cr-AGO2 and 3 (Supplementary Fig. 1d) consistent with a similar targeting mode between animal and *Chlamydomonas* proposed following our previous global analysis^1^.

As for potential slicer activity, both Cr-AGO2 and 3 possess identical catalytic tetrad residues (DEDD) required for slicer activity in the PIWI domain as At-AGO1 and HS-AGO2 (Fig. 1d)^33,36^. The conservation of these residues implies a potential ability for slicing activity for Cr-AGO2 and 3 as previously suggested^37^. However, the slicing activity could also be dependent on coordination with the N-terminal domain, as described for metazoan AGOs^13–15,38^. In Cr-AGO1, the corresponding tetrad residues are DDDD, instead of DEDD. As the difference is a conservative change between two negatively charged residues (glutamic acid to aspartic acid), it is likely that Cr-AGO1 also has potential slicing activity.

Cr-AGO2 and 3, but not Cr-AGO1 possess the hydrophobic pockets required for interaction with tryptophan-containing proteins such as GW-182 and silencing suppressors such as P38 of Turnip crinkle virus^39^ (Fig. 1e and Supplementary Fig. 1c). This feature suggests both Cr-AGO2 and 3 have the potential to recruit tryptophan-containing partners to modulate their silencing activities.

### Glycine-Arginine (GR)-enriched regions in Cr-AGOs and their prevalence in other eukaryotic AGO proteins

The sequences of all three AGOs have a predicted unstructured N-terminal extension of the N-domain. This domain is enriched in glycine and arginine (GR) residues that are involved in protein-RNA interactions due to the positively charged arginine residues^40,41^. Cr-AGO1 has a shorter GR-rich stretch of 22 amino acids (aa), while in Cr-AGO2 and Cr-AGO3 this region spans more than half the N-terminal extension of the canonical N-domain. In addition, another GR-rich stretch of ~20 amino acid is found within the canonical N-terminal domain of both Cr-AGO2 and Cr-AGO3 (Fig. 1a and Supplementary Fig. 1a).

It is likely Cr-AGO2 and 3 have similar functions due to their high degree of homology (89% aa identity to each other)^37,42^ while Cr-AGO1 is distinct from Cr-AGO2 and Cr-AGO3 (35% aa identity to both Cr-AGO2 and Cr-AGO3). Based on the domains defined by threading, the non-conservative differences between Cr-AGO2 and Cr-AGO3 are within the unstructured GR-N, the canonical N-terminal domain and the MID domain. Most differences within the GR-N are either insertion/deletions of runs of glycine (see Supplementary Fig. 1e for both conservative and non-conservative differences and Supplementary Fig. 1f for alignment of Cr-AGO2 and Cr-AGO3).

Our recent work using *Ago3* and *Dcl3* mutants demonstrated that endogenous *C*. *reinhartdii* miRNA primarily represses endogenous gene expression by targeting coding regions (CDSs) instead of 3′UTRs as in metazoan systems^1^. Therefore, we hypothesize that this CDS- specific process is dependent on an interaction of Cr-AGO3 with the ribosome via the GR-N extension^40,43,44^. The loss of the interaction between the polysome and trypanosome AGO1 by mutagenesis of the GR-N^3^ supports this model. It is possible that other AGO proteins with GR-N extension in higher plants, protozoa, and most fungi may also target coding sequence motifs in mRNAs.

To investigate the evolutionary prevalence of GR-rich N-terminal (GR-N) domains in AGOs, all 7026 AGO protein sequences from 548 species currently available in NCBI were scanned for GR-N extension throughout the whole protein sequence. For the purposes of this analysis, we defined GR-N extensions based on the occurrence of at least one contiguous region of length between 8 and 100 aa containing a minimum of 50% glycine and/or alanine residues and a minimum of 30% arginine residues. With these parameters, 991 sequences (in 252 species) were found to contain GR-N sequence. Surprisingly, most of the 991 GR-N-containing AGOs are from fungi, *Chromalveolata* and plant kingdoms, and the GR-N extensions are upstream of the N-domain (Fig. 1f and Supplementary Fig. 1g). Within the animal kingdom, a long GR-N sequence was only detected within the nematode phylum.

Many species have multiple isoforms of AGO including those either with or without the GR-N extension. For example, only AGOs that associate with 21nt miRNA in *Arabidopsis thaliana* – namely 1, 2, 3 and 5 have a GR-N extension that is >60 aa. The AGOs associated with 24-nt small RNAs, namely AGO4, 6 and 9 (Supplementary Fig. 1h) do not have these extension. The near exclusiveness of GR-N extensions to three kingdoms - fungi (except the division of Microspordia), plant and Chromalveolata - indicates that this domain is eukaryotic-specific and may have been lost during evolution of AGO proteins in complex Metazoans.

There are, however, exceptional animal PIWI-like proteins with GR-N motifs^45^ in which some of the arginines are methylated and are docking sites for the binding of various germline Tudor proteins^45–47^. The GR-N motif of Cr-AGO1 is reminiscent these PIWI GR-N motifs that are generally much shorter than the motifs in AGO proteins (Supplementary Fig. 1i,j).

### Expression and localization of the Cr-AGO proteins

**Figure 2:**
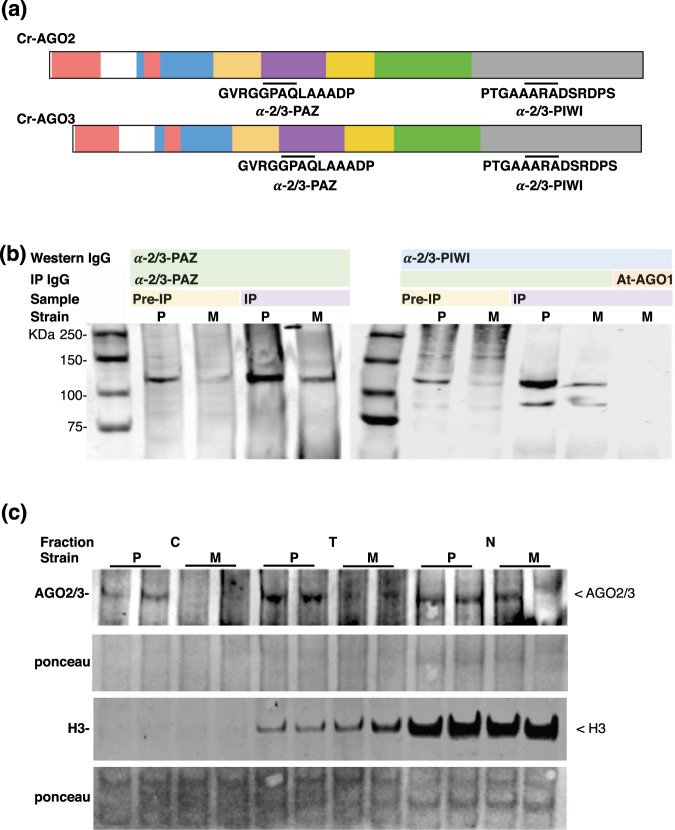
Expression and differential localisation of AGO2 and AGO3. (**a**) Antigen sites utilised for peptide-based antibody production. (**b**) Western blots for total and α-2/3-PAZ-immunoprecipitated product using extracts from either the parental (P) line or *ago3-25* (M). At-AGO1 antibody was utilized as an IP negative control. (**c**) Differential localisation of Cr-AGO2 and 3. Total protein isolated from cytoplasmic, total and nuclear fractions are indicated as C, T and N, respectively. Extracts from either Parental or *ago3-25* strain are labelled P and M, respectively. Protein bands corresponding to AGO2/3 or Histone 3 (H3) were indicated.

Based on our computational analysis, we propose that Cr-AGO2 and/or 3 rather than Cr-AGO1 are the likely candidates for miRNA and siRNA-mediated gene expression. To further characterize the Cr-AGO proteins responsible for miRNA- or siRNA-mediated gene expression, we raised antibodies against peptides from the PAZ and PIWI domains for both Cr-AGO2 and 3 (Fig. 2a), and used them for both western blot and immunoprecipitation. Due to similarity between Cr-AGO2 and 3, the antibodies raised were not able to differentiate between these proteins. Therefore we utilized a loss of function AGO3 mutant, *ago3-25* to specifically detect AGO2 protein and compare with the wild type strain, where antibodies recognized both AGO2 and AGO3 proteins. The mutant *ago3-25* was obtained in a forward genetic screen (Supplementary Fig. 2)^26^ and does not accumulate AGO3 mRNA^1^.

Both anti-PAZ and PIWI antibodies recognized a prominent band in the wild type (parental) and a less abundant band in the *ago*3-25 mutant (Fig. 2b, Supplementary Fig. 2c). In order to confirm the specificity of our antibodies, we immunoprecipitated parental and *ago3-25* mutant protein extracts with the anti-PAZ antibody. The precipitate was detected by the anti-PIWI antibody, and LC-MS/MS analysis confirmed the presence of both Cr-AGO2 and 3 in the parental strain, and Cr-AGO2 and not AGO3 in the *ago3-25* mutant (Supplementary Fig. 2 d–f). Therefore, both antibodies can specifically detect endogenous levels of Cr-AGO2 and Cr-AGO3.

To distinguish the role between Cr-AGO2 and 3, we next investigated their cellular localizations because AGO proteins in plants and animals may be either nuclear or cytoplasmic^48^. Cytoplasmic AGOs, such as At-AGO1, are normally involved in miRNA-mediated gene repression (translational repression or enhanced RNA turnover). To investigate localization of Cr-AGOs 2/3, we performed western blots from cytoplasmic- and nuclear-enriched extracts from both the parental (P) and *ago3-25* (M) strains. As expected, histone H3 was enriched in the nuclear fraction (N), while depleted from the cytoplasmic fraction (C) (Fig. 2c).

The signal corresponding to Cr-AGO-2/3 proteins was detected in the nuclear fraction of the parental strain and *ago3-25* mutant, indicating that AGO2 protein is predominantly nuclear. In contrast, we detected AGO-2/3 signal in the cytoplasmic fractions of the parental strain only. We conclude, therefore, that this signal corresponds to Cr-AGO3 (Fig. 2c, Supplementary Fig. 2g) and that Cr-AGO3 is predominantly cytoplasmic while Cr-AGO2 is mostly nuclear. We found further support for AGO3 cytoplasmic localization by assaying the presence of Cr-AGO proteins in purified polysomes. Cr-AGO-2/3 was detected in both total and polysomal fractions of parental extracts; however, this signal was less abundant from the polysomal fraction of *ago3-25* lines (Supplementary Fig. 2h). There is however a very faint band in sample *ago3-25-*polysome-C which is overall more concentrated than all other polysome samples, possibly a reflection of residual AGO2 in cytoplasm interacting with the ribosome through its GR-N domain. The cytoplasmic, polysome-associated localization of Cr-AGO3 is therefore consistent with its role in post-transcriptional regulation^1,37^.

### Cr-AGO2 and Cr-AGO3 require presence of DCL3 for stability

**Figure 3:**
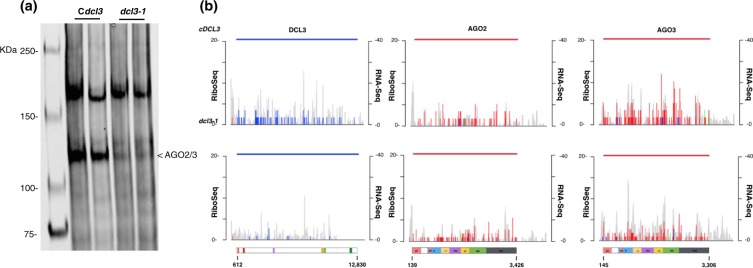
AGO2 and 3 stability is dependent on presence of DCL3. (**a**) Western blot with the antibody α-2/3-PAZ for simultaneous Cr-AGO2 and 3 detection in either *dcl3-1* or complement (C*dcl3*) background. (**b**) Ribosome profiles (coloured peaks, left axis) and corresponding RNA-seq (grey peaks, right axis) for DCL3, AGO2 and AGO3 in the *dcl3* background (bottom panels) and C*dcl3* (top panels) from previously published data^1^.

The high degree of similarity between Cr-AGO2 and 3 suggests they are associated with similar species of small RNA despite being differentially localized. To test if both Cr-AGO2 and 3 are associated with 21-nt small RNAs, we investigated their stability in a DCL3 knockout background, *dcl3-1*, in which production of 21-nt siRNA and miRNA is greatly reduced^1,26^. Figure 3a shows Cr-AGO-2/3protein abundance detected by the PAZ antibody in the *dcl3-1* mutant and in its corresponding complemented line. Similar to many reports demonstrating requirement of small RNA-association for stability in metazoan AGOs^49–51^, Cr- AGO-2/3 was significantly reduced in the *dcl3-1* mutant, suggesting that the reduction of 21-nt sRNAs prevents the accumulation of Cr-AGO2/3 proteins (Fig. 3a and Supplementary Fig. 2i). The reduction of Cr-AGO2/3 protein levels could be a consequence of a decrease in its mRNA levels or translation, or an increase in protein turnover. To differentiate these possibilities, we analyzed previous ribosome profiling and RNA-seq data^1^, which showed no significant change in the RNA abundance and translation of both Cr-AGO2 and AGO3 in the *dcl3-1* mutant, suggesting that the reduced level of these proteins in *dcl3* mutant is a consequence of a higher protein turnover (Fig. 3b and Supplementary Fig. 2j). Therefore, these results indicate that, similarly to metazoan AGO proteins, Cr-AGO2 and/or Cr-AGO3 are stabilized by association with small RNAs^49–51^.

### Cr-AGO3 but not Cr-AGO2 primarily associates with miRNA

To further explore the possibility that Cr-AGO2 and 3 bind different populations of sRNA we sequenced the RNA bound to immunoprecipitated Cr-AGO2 + 3/2 from lysates of either the parental or *ago3-25* strains to allow for comparative Cr-AGO2 and 3 small RNA analysis (Fig. 2b and Supplementary Fig. 3a).

We rationalized that, in the parental background, the IP product would be a mixture of Cr-AGO2 and 3 while, in the *ago3-25* background, the IP product would be specifically Cr-AGO2. An Arabidopsis AGO1 antibody (α-At-AGO1) was used as an IP negative control (Fig. 2b). This analysis also included total sRNA libraries from parental (P) and (M) *ago3-25* cultures. To reduce sequence bias from PCR amplification and ligation, NEXTflex adaptors containing four random terminal nucleotides were used for preparation of the small RNA libraries. It is important to utilize adaptors with random terminal nucleotides, also known as HD-tags as it has been established that conventional small RNA library preparation methods limits accurate quantification of miRNAs due to severe ligation bias^1,52–56^. The final analysis was based on the sequence reads that mapped to the *Chlamydomonas* nuclear genome (Phytozome 281). Furthermore, we also re-defined DCL3-dependent small RNA loci via segmentSeq^57^, utilizing small RNA libraries generated from both the *dcl3-1* mutant and its complement line^1,26^. The new list of loci was utilized for all siRNA analyses in this study (Supplementary Table 1) and it differs from the earlier version in that all, rather than most, miRNAs are derived from mRNA precursors^58^.

**Figure 4:**
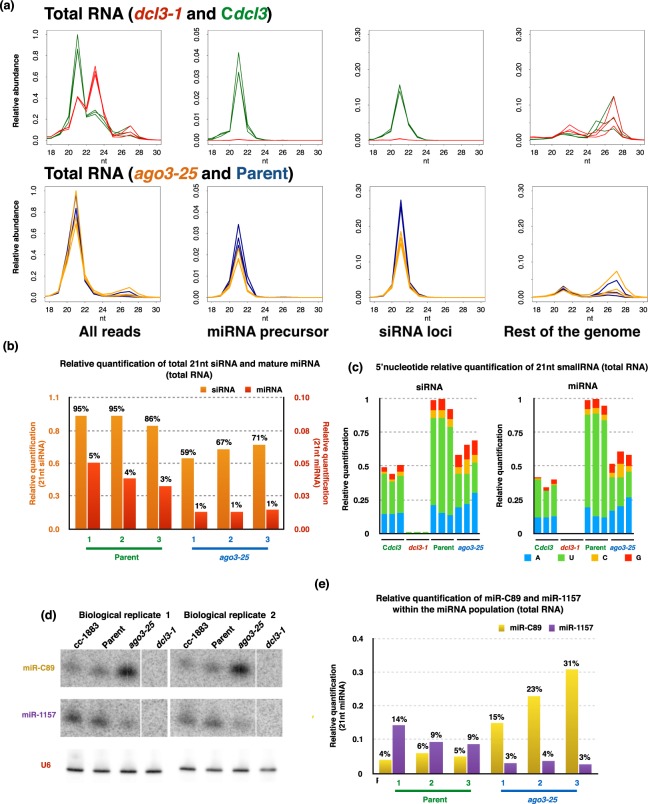
AGO3-dependent small RNAs (total RNA). (**a**) Size distributions for all small RNA-Seq libraries (normalised to library size). Top panels are total RNA libraries from *dcl3-1* (red) and complement (green) lines, while bottom panels are total RNA libraries for *ago3-25* (orange) and parental (blue) lines. The panel “all reads” represents size distribution for all reads in sequenced library. Once filtered for ribosomal and plastid RNA, reads were mapped to miRNA precursors^26^ to generate “miRNA precursor” followed by siRNA loci re-annotated in this study (“siRNA loci”) with our previously published small RNA data utilising *dcl3-1* mutant^1,26^. Remaining reads were then mapped to the nuclear genome (“Rest of the genome”). (**b**) Relative abundances of 21 nt siRNA and miRNAs (normalized to library size) in all biological triplicates for total RNA in either parental or *ago3-25* backgrounds. Percentage indicates relative abundance within the total 21nt small RNA fraction where the combination of 21nt siRNA and miRNA for biological replication 1 of Parental line scaled to 100% and the remaining libraries normalised accordingly. (**c**) 5′ nucleotide preference of total siRNA and miRNA in *dcl3-*1, C*dcl3*, Parental line and *ago3-25*. (**d**) Small RNA northern blots for miR-C89 and miR-1157 in the wild-type cc-1883, *ago3-25*, *dcl3-1* and corresponding parental line with U6 as loading control. (**e**) Relative abundances of miR-1157 and miR-C89 (normalized to library size) in all biological triplicates for total RNA in either parental or *ago3-25* backgrounds. Percentage indicates relative abundance within the total miRNA population.

Figure 4a presents the relative abundance and read-length distribution of total small RNA libraries in *dcl3-1*, complement of *dcl3-1*, *ago3-25* (M) and complemented (C) and Parental (P) lines for filtered reads, reads that maps to annotated miRNA precursors, DCL3-dependent siRNA loci, and reads that map to the rest of the nuclear genome but not annotated miRNA precursor nor siRNA loci utilized in this study.

The miRNA reads are primarily 21 nt whereas siRNA reads range from 20–22 nt with 21 nt as the primary size class. Similar to vascular plants, the abundance of siRNA in *C*. *reinhardtii* is significantly greater than miRNA and the abundance of some siRNAs and miRNAs is dependent on the presence of AGO3 (Fig. 4a,b). In addition, in correspondence with previously reported small RNA-seq in a different AGO3 mutant background, small RNAs with 5′ terminal uridine are significantly reduced while the abundance of those with 5′ terminal adenosine increased in the absence of AGO3 (Fig. 4c)^31^. The reduced concentration of miRNA and siRNA with 5′ U in the absence of Cr-AGO3 was further confirmed by northern blotting (Fig. 4d and Supplementary Fig. 3b,c and Supplementary Table 1)^26^. The miR-C89 with 5′ A, however, was more abundant in *ago3-25* than in the parent, also confirmed by northern blot (Fig. 4d,e, Supplementary Fig. 3b and Supplementary Table 2). These finding suggests that, at least in *Chlamydomonas*, stability of miRNAs with a 5′ terminal uridine is AGO3-dependent.

**Figure 5:**
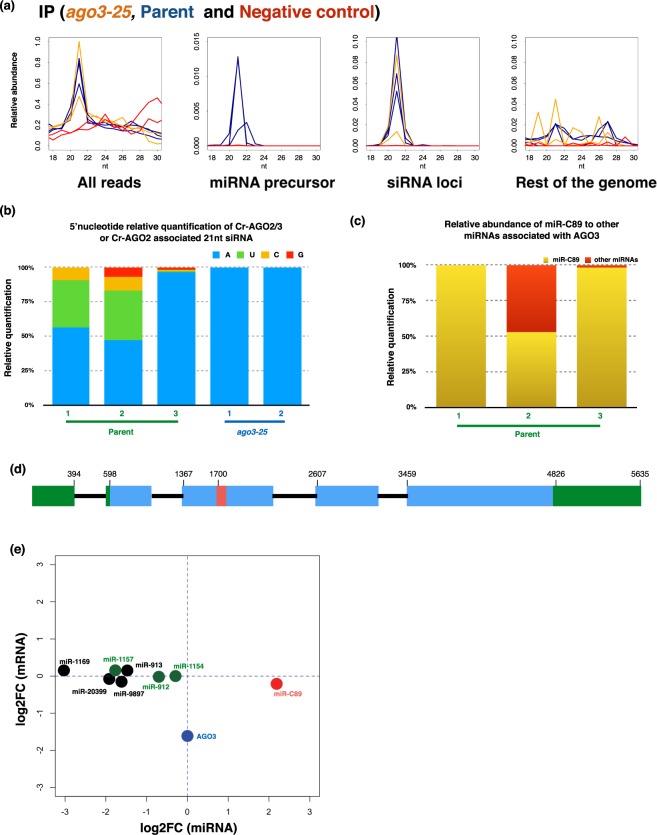
Cr-AGO2 and 3 associated small RNAs. (**a**) Size distributions for immunoprecipitated smallRNA-Seq libraries with the α-2/3-PAZ antibody in either the parental (blue) or *ago3-25* (orange) background. The negative controls, where the library was prepared from immunoprecipitated samples with the α-At-AGO1 antibody in the parental or *ago3-25* background are indicated in red. (**b**) 5′ nucleotide preference of Cr-AGO2/3 or Cr-AGO2-associated 21nt siRNA. (**c**) Relative abundance of miRC-89 in all Cr-AGO3-associated 21nt miRNA. (**d**) Schematic representation of Cre05.g239950 gene structure. Green, blue, and red boxes indicates 5′ and 3′ untranslated exons, protein coding exons and miR-C89 precursor sequence. Black line represent introns. (**e**) Correspondence between miRNA fold change (FC) between *ago3-25* and parental strain and the FC of the mRNA containing the miRNA precursor. miRNA derived from 3′UTR exons and introns are in black and green respectively while miR-C89 derived from the CDS is in red. Only the most most significantly expressed miRNA with its corresponding mRNA (parental and *ago3-25* combined) at are presented. Internal control for differential mRNA abundance – Cr-AGO3 is indicated in blue.

Figure 5a presents corresponding small RNA-Seq data of the IP in Fig. 2b. In order to enrich for the most abundant AGO2/3-associated small RNAs and minimize background, the IP was performed under high salt conditions ([NaCl] = 0.8 M) that do not impact AGO2/3 enrichment while significantly decreasing background proteins (Supplementary Fig. 2d). The absence of 21nt miRNA and enrichment of siRNA in *ago3-*25-IP background further supports Cr-AGO3 as the primary miRNA effector and both Cr-AGO3 and 2 are effectors for siRNA. It is striking that Cr-AGO2 exclusively associates with siRNA with 5′ adenosine and that miR-C89 (also with 5′ adenosine) dominates Cr-AGO3-associated miRNAs (Fig. 5b,c). The predominant enrichment of miR-C89 further supports our previous observation that this is the most prevalent endogenous miRNA for regulating gene expression under steady-state conditions^1^. Thus we conclude that the miR-C89-AGO3 complex is the primary endogenous miRNA complex for post-transcriptional gene regulation in *Chlamydomonas*.

It is striking that miR-C89 with a 5′ adenosine accounts for most of the miRNA bound to Cr-AGO3 corresponding to it being the most abundant miRNA in our sRNA datasets^1,26^. This finding differs from previous studies showing miRNA bound to Cr-AGO3 with a 5′ uridine^58^. The difference is likely due, in part, to (1) our utilization of adaptors with randomized terminal nucleotides to reduce biased ligation to small RNA with 5′ uridine and PCR duplication of reads^52^ and (2) the stringent IP condition (NaCl = 0.8 M) so that only the most abundant or tightly bound AGO2/3-associated small RNAs would be retained. Our isolation of small RNA from endogenous instead of over-expressed tagged-AGO ensures capturing of biologically relevant interactions between functional AGO and small RNAs and may also contribute to the difference in our dataset and that of Voshall *et al*. (2015).

miR-C89 is unique amongst the miRNAs in that the precursor structure is embedded within the coding region of a gene (Cre05.g239950) (Fig. 5d). All other miRNAs are derived from non coding regions of coding mRNAs including introns^1,26^. Cre05.g239950 is more abundant in the *dcl3-1* background (Supplementary Fig. 4 of Chung *et al*. 2017) consistent with its stability being inversely affected by processing of miRNA precursor^1^. In contrast, however, similar to other miRNA-containing genes, the level of Cre05.g239950 is not significantly affected by presence nor lack of AGO3 suggesting Cr-AGO3 is unlikely to be involved in miR-C89 processing (Fig. 5e and Supplementary Fig. 3d).

To explain the higher level of miR-C89 in *ago3-25* background than in its wild type parent we propose that turnover of this miRNA is accelerated when it is associated with AGO3 (Figs 4d,e and 5e). Further analysis of the contrasting properties of miR-C89 and other miRNAs in association with Cr-AGO3 will be informative about the biology and molecular biology of RNA silencing in *C*. *reinhardtii*.

## Materials and Methods

### Culture and sample preparation

Fresh, independent colonies of *Chlamydomonas reinhardtii* cells (CC-1883) were cultured and maintained as previously described^1^. For IP, lysates were prepared by harvesting cells in mid-log phase (OD_750_~0.6). Cell cultures were incubated on ice for 30 min prior to centrifugation at 4000 rpm, 5 min, 4 °C. The pellet was re-suspended in IP lysis buffer (1 × PBS, NaF 50 mM, NaSVO_4_ 1 mM, MgCl_2_ 5 mM, PMSF 1 mM, NP40 0.1%, DTT 5 mM, NaCl 0.8 M, 1X protease inhibitor cocktail without EDTA (Roche)). The resuspended cells were then dounce homogenised on ice 20×, followed by centrifugation at 4700 rpm, 4 °C, 30 min. The clarified samples were either snap frozen in liquid nitrogen or 10% TCA-acetone precipitated and resuspended in resuspension buffer (Urea 8 M, NaCl 0.5 M and DTT 5 mM) for western blot analysis.

### Immunoprecipitation and small RNA library preparation

Two antibodies α-2/3-PAZ: GVRGGPAQLAAADP and α-2/3-PIWI: PTGAAARADSRDPS (GenScript and Agrisera, AS142799) were raised for all immuno-detection analyses. All western blots were performed with 1/500 overnight 4 °C incubation with the primary antibody and 1/5000 anti-rabbit for 1 h at room temperature prior to development. All immunoprecipitations were performed with antibodies conjugated to tosylactivated streptavadin beads M280 (Invitrogen) and immunoprecipitation was performed following the manufacturer’s protocol after the last wash step, the beads were spilt in half for either directly boiling in SDS-PAGE sample buffer for western blot or total RNA extraction followed by small RNA library preparation as described previously^1^.

### RNA extraction and small RNA northern blot

RNA was extracted from cell cultures grown to a cell density of 2–3 × 10^6^ cells/ml in TAP media, at 23 °C and under constant light. RNA extraction was performed as previously described^59^, with the following modifications: RNA was extracted from live cells after centrifugation of the culture, without prior freezing; PureZol (Biorad) was used instead of Trizol and volumes were scaled down to perform extraction in 2 ml tubes. RNA quality was assessed in gel and quantify in Nanodrop (ThermoFisher Scientific).

Small RNA detection by Northern blot was performed as described in^59^ (http://www.plantsci.cam.ac.uk/research/davidbaulcombe/methods/downloads/smallrna.pdf/view). 15 µg of total RNA was separated in 15% TBE-Urea gels (Invitrogen) and transfer by capillarity to an Amersham Hybond N+ membrane (GE Healthcare). A DNA oligo corresponding to the reverse complementarity sequence of the small RNA to assay was radiolabeled with γ^32^P-ATP (PerkinElmer)by the action of the T4 polynucleotide kinase (ThermoFisher Scientific), and used to probe membranes with the immobilized RNA samples. Radioactive signals were detected using a storage phosphor screen (GE Healthcare), which was scanned using a Typhoon 9400 imager (GE Healthcare). For detection of U6 RNA species, used as a loading control, a fluorescent-labeled DNA oligo was employed, and blots were scanned using an Odyssey Imaging System (LI-COR). Images were analyzed using the ImageJ software.

Oligo sequences used for small RNA northern blot: miR-C89: 5′CATTCCACACTTTTCACGCCT3′, miR1157: 5′CACCTGGTCCCGCTACCTGAA3′ and U6: 5′IRD800/TAACAGGTTATGAGCCCCGG3′

### Differential isolation of proteins

Cells were similarly harvested as for IP lysates except resuspended cells were subjected to 2× freeze thaw cycle followed by centrifugation at 14000 rpm, 5 min, 4 °C. The Pellet was washed 3× with 1 ml IP lysis buffer containing NP40 0.5% followed by TCA-acetone precipitation for total nuclear protein extraction. The supernatant was also TCA-acetone precipitated for total cytoplasmic protein extraction.

Total polysome protein was obtained by addition of 100 µg/ml cycloheximide and 100 µg/ml chloramphenicol to 100 ml cells in mid-log phase for 5 min followed by incubation in ice for 10 min and pelleted at 4700 rpm for 1 min at 4 °C. The cell pellet was resuspended in 1 ml ice cold polysome buffer (Tris-Cl pH 7.5 10 mM, KCl 140 mM, MgCl_2_ 5 mM, DTT 0.5 mM, NP40 0.5%, TritonX100 1%, PMSF 1 mM, NaF 50 mM, NaSVO_4_ 1 mM, 1X roche protease inhibitor without EDTA cocktail, 100 µg/ml cycloheximide and 100 µg/ml chloramphenicol and SUPERase Inhibitor 10 U/ml, TurboDNase 5 U/ml). The resuspension was passed through 30 G needle twice, followed by centrifugation at 14000 rpm, 10 min, 4 °C. The supernatant was layered on 8 ml of 35% sucrose made in polysome buffer and centrifuged at 38000 rpm, 18 h, 4 °C in a SW41 rotor. The pellet was then TCA-acetone precipitated for western blot analysis. Anti-histone3 antibody (Agrisera #AS15 2855) and Anti-ribosomal protein S3 (Cell Signalling #2579) were utilised to assess enrichment of nuclear or cytoplasmic extract.

### Mass spectrometry of Cr-AGO2 and AGO3

1D gel bands were transferred into a 96-well PCR plate. The bands were cut into 1 mm^2^ pieces, destained, reduced (DTT) and alkylated (iodoacetamide) and subjected to enzymatic digestion with chymotrypsin overnight at 37 °C. After digestion, the supernatant was pipetted into a sample vial and loaded onto an autosampler for automated LC-MS/MS analysis.

All LC-MS/MS experiments were performed using a Dionex Ultimate 3000 RSLC nanoUPLC (Thermo Fisher Scientific Inc, Waltham, MA, USA) system and a Q Exactive Orbitrap mass spectrometer (Thermo Fisher Scientific Inc, Waltham, MA, USA). Separation of peptides was performed by reverse-phase chromatography at a flow rate of 300 nL/min and a Thermo Scientific reverse-phase nano Easy-spray column (Thermo Scientific PepMap C18, 2 µm particle size, 100 A pore size, 75 µm i.d. × 50 cm length). Peptides were loaded onto a pre-column (Thermo Scientific PepMap 100 C18, 5 µm particle size, 100 A pore size, 300 µm i.d. × 5 mm length) from the Ultimate 3000 autosampler with 0.1% formic acid for 3 minutes at a flow rate of 10 µL/min. After this period, the column valve was switched to allow elution of peptides from the pre-column onto the analytical column. Solvent A was water + 0.1% formic acid and solvent B was 80% acetonitrile, 20% water + 0.1% formic acid. The linear gradient employed was 2–40% B in 30 minutes.

The LC eluant was sprayed into the mass spectrometer by means of an Easy-Spray source (Thermo Fisher Scientific Inc.). All *m/z* values of eluting ions were measured in an Orbitrap mass analyzer, set at a resolution of 70000 and was scanned between *m/z* 380–1500. Data dependent scans (Top 20) were employed to automatically isolate and generate fragment ions by higher energy collisional dissociation (HCD, NCE:25%) in the HCD collision cell and measurement of the resulting fragment ions was performed in the Orbitrap analyser, set at a resolution of 17500. Singly charged ions and ions with unassigned charge states were excluded from being selected for MS/MS and a dynamic exclusion window of 20 seconds was employed.

Post-run, the data was processed using Protein Discoverer (version 2.1., ThermoFisher). Briefly, all MS/MS data were converted to mgf files and the files were then submitted to the Mascot search algorithm (Matrix Science, London UK) and searched against a customised NCBI *Chlamydomonas reinhardtii* database (14491 sequences; 6575547 residues) and a common contaminant sequences (115 sequences, 38274 residues). Variable modifications of oxidation (M), deamidation (NQ) and carbamidomethyl were applied. The peptide and fragment mass tolerances were set to 5 ppm and 0.1 Da, respectively. A significance threshold value of p < 0.05 and a peptide cut-off score of 20 were also applied.

### Computational analysis

To define regions of AGO containing GR-N extension, 7026 currently available AGO protein sequences were extracted from the refeseq database in NCBI and scanned for GR-N extension using a combination of C-shell C++ scripts. The scanning parameter for GR-N extension were based on the occurrence of at least one contiguous region of length between 8 and 100 aa containing a minimum of 50% glycine and/or alanine residues and a minimum of 30% arginine residues. The purpose to include alanine is based on the hypothesis that the key interactor is the positively charged arginine residue which is positioned by small, uncharged restudies such as glycine and alanine. The output was then post-processed by a combination of C-shell scripts and the statistical package R. The phylogenic tree were produced by FigTree (v1.4.2)^60^.

Differential expression of mRNA were performed using DE-Seq2 and Small RNA data processing were performed as described in Chung *et al*. 2017. The software package segmentSeq was utilized for identification and differential expression analysis of 21-nt DCL3-dependent small RNA loci^57^. The reference genome and transcriptome of *Chlamydomonas reinhardtii* used were Phytozome v5.0 and 281, respectively.

## Supplementary information

Supplementary information

## Data Availability

All raw sequencing data are deposited on Arrayexpress with accessions E-MTAB-5726. All code used for bioinformatic analysis is available on request.
