# Supplementary material for: Distinct roles of Argonaute in the green alga Chlamydomonas reveal evolutionary conserved mode of miRNA-mediated gene expression
Source: Sci Rep. 2019 Jul 31;9:11091. doi: 10.1038/s41598-019-47415-x (PMC6668577; doi:10.1038/s41598-019-47415-x)
Supplement: Supplementary file 1 — Supplementary information [file 41598_2019_47415_MOESM1_ESM.pdf]

SUPPORTING INFORMATION

Distinct roles of Argonaute in the green alga *Chlamydomonas* reveal evolutionary conserved mode of miRNA-mediated gene expression.

Betty Y-W. Chung*[1, 2], Adrian Valli[1, 3], Michael J. Deery[4], Francisco J. Navarro[1], Katherine Brown[2], Silvia Hnatova[1], Julie Howard[4], Attila Molnar[5] and David C. Baulcombe*[1].

[1] Department of Plant Sciences, University of Cambridge, Cambridge, CB2 3EA, United Kingdom
[2] Department of Pathology, University of Cambridge, Cambridge, CB2 1QP, United Kingdom (present address)
[3] Department of Plant Molecular Genetics, Spanish National Centre for Biotechnology, Madrid, 28049, Spain (present address)
[4] Cambridge System Biology Centre and Department of Biochemistry, University of Cambridge, CB2 1GA, United Kingdom
[5] Institute of Molecular Plant Sciences, University of Edinburgh, Edinburgh, EH9 3BF, United Kingdom

*** Correspondence should be addressed to:**
Betty Y-W. Chung, bcy23@cam.ac.uk, Tel:(+44)1223-333331
David C. Baulcombe, dcb40@cam.ac.uk, Tel:(+44)1223-339386

Supplementary Figure 1: Structural features of *Chlamydomonas* Argonautes

(a) Cr-AGO protein sequences were extracted from Phytozome V5. GR-rich domains are highlighted in pink, N-terminal domains in cyan, L1 in orange, PAZ in purple, L2 in yellow, MID in green and PIWI in grey. All domains are defined by threading using the crystalised Human AGO2 as template – PDB:4W5T [32].

(b) Modelling of Cr-AGO MID domain structure to At-AGO1 MID domain. Homology model of the MID domain of all Cr-AGOs, superimposed in the crystal structure of the At-AGO1 MID domain complexed with UMP. Only the modelled UMP is shown for the Cr-AGO model for clarity. The protein side chain corresponding to residue N687 in At-AGO1 is shown as sticks in magenta and the two surrounding residues are coloured in red. The highly conserved 5' phosphate recognition residues are shown as sticks in yellow. The At-AGO1 structures were derived from PDB:4G0P, where the UMP was modelled into crystallized At-AGO2 and At-AGO5 structures (PDB:4G0M and 4G0O respectively).

(c) Tandem tryptophan-binding pockets in the PIWI domain. Closed-up view of surface representation showing the tryptophan-binding pockets in human AGO2, *Arabidopsis* AGO1, 4, 6 and 9 and *Chlamydomonas* AGO 1, 2 and 3. Residues forming the binding pockets, that are identical or have conservative changes relative to human AGO2, are highlighted in blue. Residues showing non-conservative changes are highlighted in magenta. All structures are models based on the human AGO2 crystal structure (PDB:4W5O). Bottom panel is the thirty degree anti-clockwise rotated view for clearer representation of pocket 1.

(d) Homology modelling of Cr-AGO2 complexed with miRNA:gRNA duplex (Phyre 2). The α-carbon for non-conservative and conservative substitutions are indicated with red and yellow spheres respectively. Many of the non-conservative differences are located near loop regions, except within the MID domain.

(e) Alignment of Cr-AGO2 and Cr-AGO3. The GR-rich domains are highlighted in pink, N-terminal domain in cyan, L1 in orange, PAZ in purple, L2 in yellow, MID in green and PIWI in grey. The alignment was performed using ClustalOmega.

(f) Figure 1f in higher resolution.

(g) Annotation of GR-N extensions for Arabidopsis Argonautes. At-AGO protein sequences were extracted from TAIR 10. Glycine and arginine residues are highlighted in yellow and red, respectively.

(h) As in panel (b) except that only species containing germline-specific PIWI proteins are shown. Species with or without N-terminal GR-N domain are highlighted in blue and green, respectively.

(i) Length distribution of AGO and PIWI G/AR-rich domains.

Supplementary Figure 2:

(a) Mapping of their corresponding insertional mutagens by RESDA-PCR localized the insertions into Cre16.g689647 (Ago3) [26].

(b) *ago3-25* complemented with Ago3 genomic DNA display the light-dependent lethal phenotype under nitrate induction of artificial miRNA against the PSY gene [26].

(c) Enlarged version of Figure 2b with corresponding Ponceau stain.

(d) Immunoprecipitated Cr-AGO2/3 with α-2/3-PAZ (i.e. in the parental background) in a gradient of NaCl concentrations (0.15M, 0.3M, 0.5M and 0.8M). The western blot was probed with the antibody α-2/3-PIWI and the resulting purified protein at ~110kDA was used for LC-MS-MS.

(e) Peptides detected by mass spectrometry confirming the presence of Cr-AGO2 in the α-2/3-PAZ IP product. Sequences in red are peptide matches that correspond to Cr-AGO2 protein sequence.

(f) Peptides detected by mass spectrometry confirming the presence of Cr-AGO3 in the α-2/3-PAZ IP product. Sequences in red are peptide matches that correspond to Cr-AGO3 protein sequence.

(g) Independent repeat for differential localisation of Cr-AGO2 and 3. Total protein isolated from cytoplasmic and nuclear fractions are indicated as C and N, respectively. Extracts from either Parental or *ago3-25* strain are labelled P and M, respectively. Protein bands corresponding to AGO2/3 are indicated.

(h) Enrichment of Cr-AGO3 in purified polysome fraction from either *ago3-25* or respective parental strain. Anti-histone3 antibody (Agrisera) and Anti-ribosomal protein S3 (Abcam) were utilised to validate nuclear or cytoplasmic extraction.

(h) Figure 3a with corresponding Ponceau stain to reflect total protein loading.

(i) Histogram of 5' end positions of normalized RPF (colored, left-axis) and RNA-Seq (grey, right-axis) 27-nt reads mapped to Cr-AGO2 and 3 transcripts, respectively, in all biological replicates. The red horizontal line indicates the CDS. Data derived from pervious work [1]

Supplementary Figure 3:

(a) Histone 3 Western blot of either total protein (T) or protein extracted from the supernatant (S) used for Cr-AGO2/3-IP.

(b) Small RNA northern blot for miR-C89 and miR-1157 in the wild-type cc-1883, two independent Parental lines, *ago3-25*, two independent lines of *ago3-25* complements and *dcl3-1.* U6 was utilised as loading control.

(c) Small RNA Northern blot for miR-1162, 1157, 9897 in two independent *ago3*-25 complement lines, biological replicates of *ago3-25* and the parental line. U6 was utilised as loading control.

(d) Volcano plot for all differentially expressed mRNAs between parental and *ago3-25* in duplicates. *y-axis* represents significance of differential expression using DE-Seq2. All grey dots represents a single mRNA while AGO3 is highlighted in blue, genes containing miRNA precursor within 3'UTR exons or introns in black and green, respectively and Cre05.g239950, the mRNA that also codes for miR-C89 in red. Horizontal dotted-line indicates significance of 0.01.

Supplementary Table 1:
Normalised siRNA abundance.

Supplementary Table 2:
Normalised miRNA abundance (Total RNA).

Supplementary Table 3:
Normalised miRNA abundance (AGO-IP).

Supplementary Figure 1

(a)

>Chlamydomonas reinhardtii|Argonaute 1|Cre02.g141050.t1.1
MLSKVQLPRRAGRANSATPVSARYSDSANRPGGRGGGQPGRGGGGRGGGSGRGSSSSTAPKELLTPAQYWPSFWTRLFTGLPLG
PVEPRQLANGHAARPGYSAAGKTIQVLTNHVPLNVGVRTVHQYDVRISRVANSAPTSSAAAPSGQGGGKPGRGRKEAKVSGQEA
QAVLVALLGDTGAGGSGAAGGVWVFDGSQALYGVEGDLRRRAAAAAAGAGGAGGDGELQVQLAAAPTTGVVGGTYKVDLKLVGA
LDVAECLREAAQMPGPTTRRPPPDEEQPEGSPAPAGVLPPPPAPPATSGAAALAVLDLVTRAGVAWDVASVRTGGGSYVSYKQE
DASGPVKGLLPMKLLRGFRSAVAQVAAGTTLTIDAASAVVAGDGPLLGQLQAALAAGRDVSALATELGNSKNARRLEEALVGLE
VQPSYTANRYKLSGKLGKSARSLAFEFEGRKISVQTYFKERYGVSLQHPDLPCVLDKKGGALPIELLTVVKYQKRMSRLDARQK
ADYIRTAALKPADRMRAMREALTRQLGSVRGQVARAFGLAPFNDMPDFLKVPAQLLPGPLLQVAKQQAVEPGNKGEWAGGGVAV
APEWRSGAVLCYLSQSEGQAPLSGLLAALSKALSASSASALPPNIPVIWASLPKPGMSPDKAAADAEFWLNRAAAKAQEQYKRQ
AQMVMVVLPDRGGVGLYDAIKAAGDGKLGVATQCVNPAKAGLVGPKASSGPSFSYAAQLALSVNAKLGGATTRPAGRPNDWLPL
LGGRRLMVLGADISRGAAAALSGRGGDQQLAAAEAESEAQVSAGVEYAAVVGSADSHAVDYRVQLSAQVAGNRDIVVSMRESVR
RLLLQYGLPQGGAVAAGGPRPSSELPEVVLMYRDGLSESQFDRALAEEFTAIVQACADVGGPSYRPRICFVVVQKSHNTRFFPT
SAESTHKSGNVLPGTAVDRGVTDPHAFDFFLNSHAGIQGTNRAPRYTVLVDEVGFTAEALQLLTHTLCHTYPACTRALSLPPPV
RYADRAADRARTLRYMVPRPSGKQGRGVDWYQMLPTLPHAGHVV

>Chlamydomonas reinhardtii|Argonaute 3 |Cre16.g689647.t1.1
MSGRGRGGGGGYGGGGGYGGGGGGGRGGGGGDRGGGGYGGGGRGGGGGGGYGGGGGGGGRGGGGGGGYGGGGGRGGGGGGGGGG
VVSPAQAAASLDAILKSAKSLTAPVKVETTADGRPLGITRRPSAGSVGKAVALLANYFALATTPAFPGQAYHYDVEIRSVETPG
GGGGGGRGGGRGGGRGGGRGGRGGAAPAPATAEESAGAPAAEAGGEDLPPRLAHRVMASAAAAHGWPAGAWRFDGRKNLFLPGE
LLPREVREWPVTLKPREGDKSERDKGFVVTTKWAACVGLTQLQDYLARRQQTAPRDAMQVLDIVIRHAFAIDPRCTVVGRGFYY
GGEGVMPLGGGAEVWSGFQQSFKAVQAGLTLNLDSSFAAFMSARPLPELLAEGAGVRGGPAQLAAADPHRLRAAARGLAGFKVE
FPMPGGRARRKALVGLSEQGADRTMFMNEKEGREMSVAEYFRSTGRPLRHPGLPCANVGDRRRAVFIPVELCTVVAGQRRMKLD
ATQSAGMITAAKQDPAVKKEACDKQAKRVAEALAAGGTDRCWGLKLATGMLPVQGRVLPNPVLQYGDPQAFDAGPLGSWNLRNV
KFVDPRALDSWGVAVMMNQADVDFDGDNSLWQFLEDLTGDMIRCGMRVASPVTAASTDSPPVEFGGMPGGGGRGGGRGIEATMR
AAADAAAARYKKPAQLVLVVLPEKTADEYREVKRVSDIELGIPSQVVVASKARVGYRAHKGGGPQYCANVAMKINNKLGGVNVQ
LSGGLRNMPVLGGAGAVPFMVLGADVTHPTGAAARADSRDPSVAAVVASLDASLGRWASRVLLQAGRQEVITGMCGATKELLLE
FYRANKQVKPQRLVMYRDGVSEGQFEQVLAEEYTALRRACRELEEGYRPAITFVVVQKRHNTRLLPSDRAASDQKGNVVPGTVV
DSGITAPDGFDFYLNSHAGLQGTNKPAHYHVLVDEIGFGADGIQLLTYWLCYLYQRTTKSVSYCPPAYYADRAAFRGRTLLAAS
SSASDSASETASRSGRGAGAAEGGASAPPTFAGIHRNLTNVLYFM

>Chlamydomonas reinhardtii|Argonaute 2 alt|Cre04.g214250.t2.1
MSGRGRGGGGGYGGGYGGGGGGSRGGGGGGGSGGGDRGYGGGGGGRGGGGYGGGSAGGGGYGGGGGRGGGGGGGGYGGGGGGRG
GGGGGGGVVSPQQAAATLDVVLKSARTLTAKVNVETTPDGRPLGITRRPNAGTVGKAVALLANYFALATTPAFPGQAYHYDVEI
RSVEEAAGGGGGGGRGGGRGGGGGGGGGRGGRRDLAPASATGDVPGGAAAAAAAEDLPPRLAHRVMAAAAAAHGWPAGAWRFDG
RKNLFLPGELLPREVREWPVTLKPREGDKSERKGFVVATKWAACVGLTQLQDYLAQRQQTAPRDAMQVLDIVIRHAFAIDPRCT
VVGRGFYYGGEGVMPLGGGAEVWSGFQQSFKAVQAGLMLNLDSSFAAMSARPLPELLAEGAGVRGGPAQLAAADPRRLRAAARS
LVGFKVEFPMPGGRARRKMLTGLSEQGADRTMFMNEKEGREMSVAEYFRSTGRPLRHPGLPCANVGDRRRAVYIPLELCTVVAG
QRRMKLDATQSAGMITAAKQDPAVKAEACNKQAKRVAEALAAGGTERSWGLKLGTGMLPVQGRVLPNPVLQYGNRQDFDAGPLG
SWNTLNVKFVDARALDSWAVAVMMNQADVDFDGDNSIWKFLEDLCSAMITRGMRVASPVTRGSNDSPPVEYGGAAAGGGAAGRG
GGARGVEATLRAAADAAAARYKKPAQLVLVVLPVKPSDEYREVKRVSDIELGIPSQVVVGGKARVGYREQRGGGPQYCANVAMK
INNKLGGVNVQLSGGLRYMPVLGGAGSVPFMVLGADVTHPTGAAARADSRDPSVAAVVGSLDASLGRWASRVLLQAGRQEVITG
MCGATKELLLEFYRANKQVKPQRLVMYRDGVSEGQFEQVLAEEFTALRRACRELEEGYRPAITFVVVQKRHNTRLLPNDRASAD
PKGNVVPGTVVDSGITAPDGFDFYLNSHSGLQGTNKPAHYHVLVDEIGFGADGMQLLTYWLCYLYQRTTKSVSYCPAAYYADRA
AFRGRTLLAASSSASDSASESGSRAGRGAGAAEGGASAPPTFAGIHRNLSNVLYFM

(b)

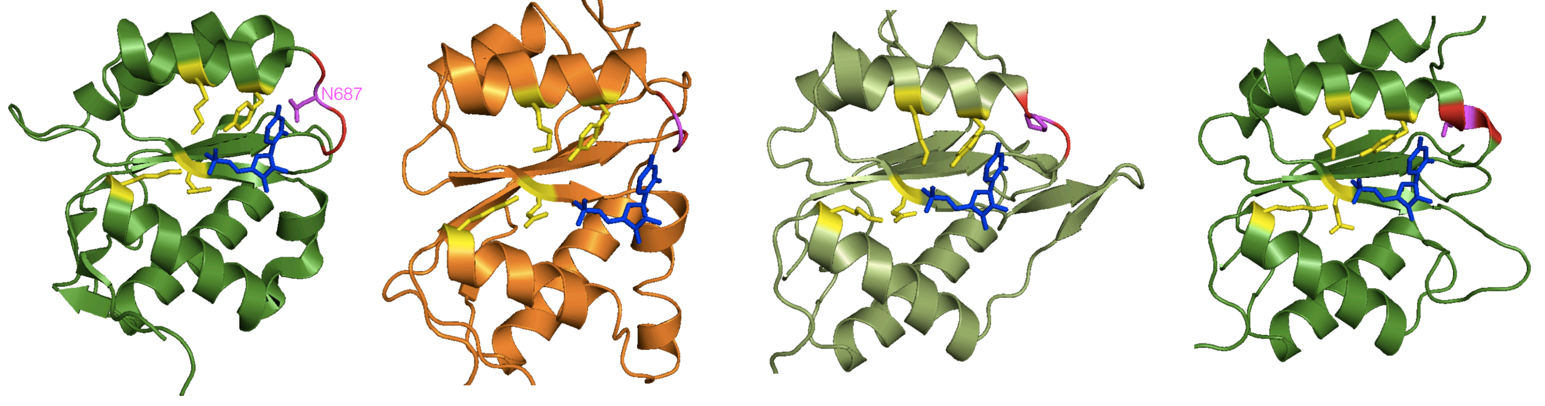

At-AGO1 Cr-AGO1 Cr-AGO2 Cr-AGO3

(c)

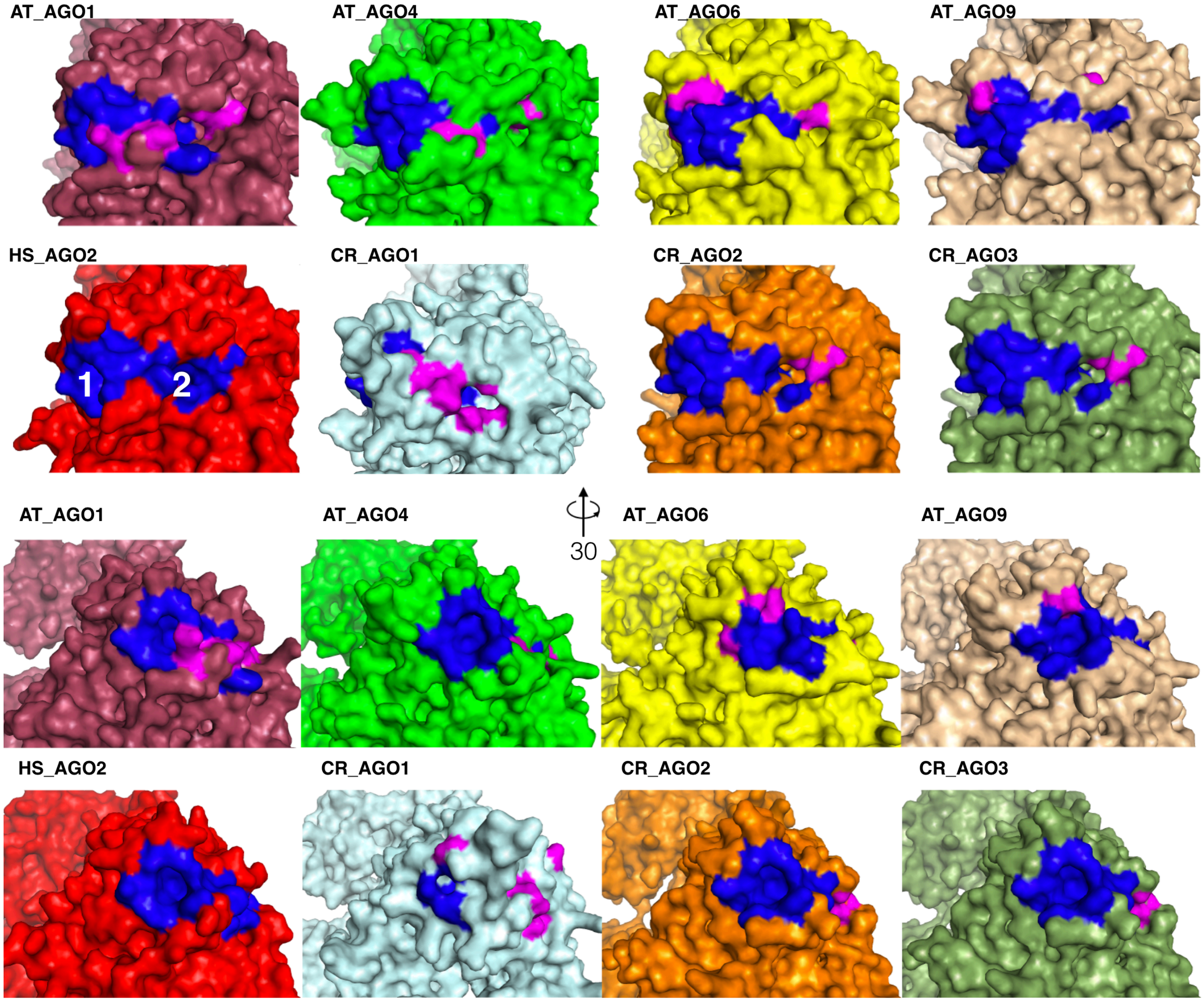
AT_AGO1
AT_AGO4
AT_AGO6
AT_AGO9
HS_AGO2
1
2
CR_AGO1
CR_AGO2
CR_AGO3
AT_AGO1
AT_AGO4
30
AT_AGO6
AT_AGO9
HS_AGO2
CR_AGO1
CR_AGO2
CR_AGO3

(d)

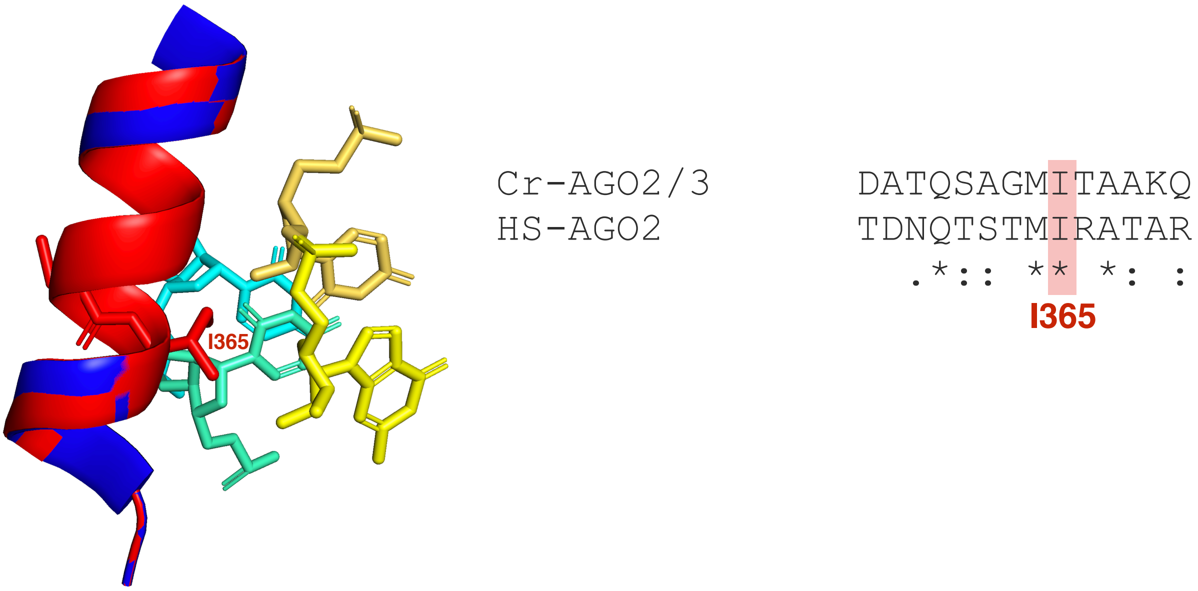
I365
Cr-AGO2/3 DATQSAGMITAAKQ
HS-AGO2 TDNQTSTMIRATAR
.*:: ** *: :
I365

(e)

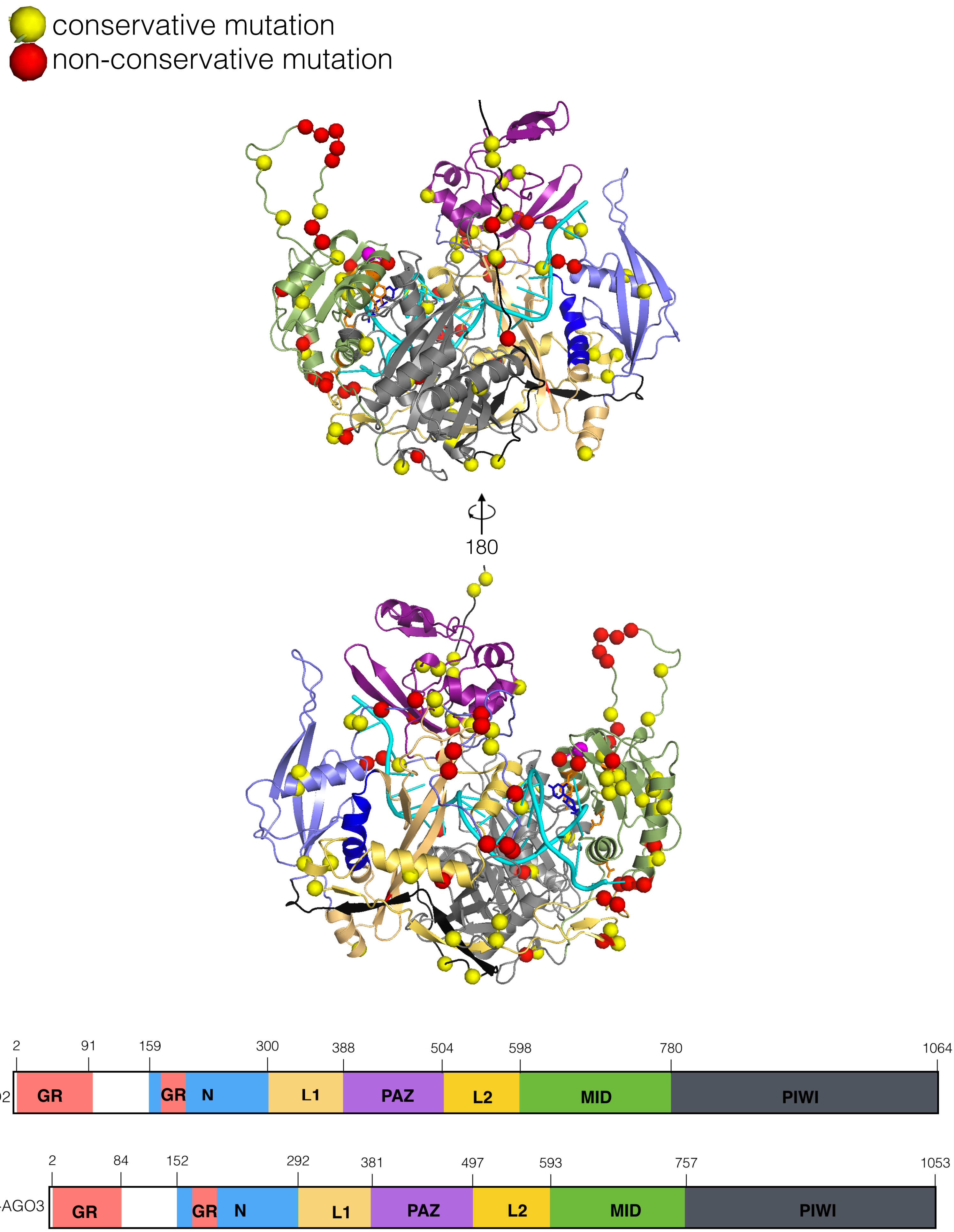

conservative mutation
non-conservative mutation
180
Cr-AGO2
2
91
159
300
388
504
598
780
1064
GR
GR
N
L1
PAZ
L2
MID
PIWI
Cr-AGO3
2
84
152
292
381
497
593
757
1053
GR
GR
N
L1
PAZ
L2
MID
PIWI

(f)

```
CR_AGO2    MSGRGRGGGGGYGGG--YGGGGGGSRGGGGGGGSGGDRGYGGGGG-GRGGGGYGGGSAG
CR_AGO3    MSGRGRGGGGGYGGGGGYGGGGGGGRGGGGGDRGG---GGYGGGGRGGGGGGYGGG---
           ***************  *******.******   .*     ******   *  ********

CR_AGO2    GGGYGGGGGRGGGGGGGGYGGGG-GGRGGGGGGGGVVSPQQAAATLDVVLKSARTLTAKV
CR_AGO3    ----GGGGGR-GGGGGGGYGGGGGRGGGGGGGGGGVVSPAQAAASLDAILKSAKSLTAPV
               ****** ************  * ************ ****:**.:****::*** *

CR_AGO2    NVETTPDGRPLGITRRPNAGTVGKAVALLANYFALATTPAFPGQAYHYDVEIRSVEEAAG
CR_AGO3    KVETTADGRPLGITRRPSAGSVGKAVALLANYFALATTPAFPGQAYHYDVEIRSVETPGG
           :**** ************.**:***************************************   .*

CR_AGO2    GGGGGGRGGGRGGGGGGGGGGRGGRRDLAPASATGDV-PGGAAAAAAAEDLPPRLAHRVMA
CR_AGO3    G-GGGGRGGGRGGGRG--GGRGGRGGAAPAPATAEESAGAPAAEAGGEDLPPRLAHRVMA
           * *********** *   ******    *** **.:    *. ** *..*************

CR_AGO2    AAAAAHGWPAGAWRFDGRKNLFLPGELLPREVREWPVTLKPREGDKSER-KGFVVATKWA
CR_AGO3    SAAAAHGWPAGAWRFDGRKNLFLPGELLPREVREWPVTLKPREGDKSERDKGFVVTTKWA
           :************************************************ *****:****

CR_AGO2    ACVGLTQLQDYLAQRQQTAPRDAMQVLDIVIRHAFAIDPRCTVVGRGFYYGGEGVMPLGG
CR_AGO3    ACVGLTQLQDYLARRQQTAPRDAMQVLDIVIRHAFAIDPRCTVVGRGFYYGGEGVMPLGG
           *************:**********************************************

CR_AGO2    GAEVWSGFQQSFKAVQAGLMLNLDSSFAA-MSARPLPELLAEGAGVRGGPAQLAAADPRR
CR_AGO3    GAEVWSGFQQSFKAVQAGLTLNLDSSFAAFMSARPLPELLAEGAGVRGGPAQLAAADPHR
           ******************* ********* ****************************:*

CR_AGO2    LRAAARSLVGFKVEFPMPGGRARRKMLTGLSEQGADRTMFMNEKEGREMSVAEYFRSTGR
CR_AGO3    LRAAARGLAGFKVEFPMPGGRARRKALVGLSEQGADRTMFMNEKEGREMSVAEYFRSTGR
           ******.*.**************** *.********************************

CR_AGO2    PLRHPGLPCANVGDRRRAVYIPLELCTVVAGQRRMKLDATQSAGMITAAKQDPAVKAEAC
CR_AGO3    PLRHPGLPCANVGDRRRAVFIPVELCTVVAGQRRMKLDATQSAGMITAAKQDPAVKKEAC
           *******************:**:********************************* ***

CR_AGO2    NKQAKRVAEALAAGGTERSWGLKLGTGMLPVQGRVLPNPVLQYGNRQDFDAGPLGSWNTL
CR_AGO3    DKQAKRVAEALAAGGTDRCWGLKLATGMLPVQGRVLPNPVLQYGDPQAFDAGPLGSWNLR
           :***************:*.*****.*******************:  * **********

CR_AGO2    NVKFVDARALDSWAVAVMMNQADVDFDGDNSIWKFLEDLCSAMITRGMRVASPVTRGSND
CR_AGO3    NVKFVDPRALDSWGVAVMMNQADVDFDGDNSLWQFLEDLTGDMIRCGMRVASPVTAASTD
           ****** ******.*****************:*:****** . **  *********  .*.*

CR_AGO2    SPPVEYGGAAAGGGAAGRGGGARGVEATLRAAADAAAARYKKPAQLVLVVLPVKPSDEYR
CR_AGO3    SPPVEFGGMPGGGGR----GGGRGIEATMRAAADAAAARYKKPAQLVLVVLPEKTADEYR
           *****:**   .***    **.**:***:*********************** *  :****

CR_AGO2    EVKRVSDIELGIPSQVVVGGKARVGYREQRGGGPQYCANVAMKINNKLGGVNVQLSGGLR
CR_AGO3    EVKRVSDIELGIPSQVVVASKARVGYRAHKGGGPQYCANVAMKINNKLGGVNVQLSGGLR
           ******************..*******  ::*********************************

CR_AGO2    YMPVLGGAGSVPFMVLGADVTHPTGAAARADSRDPSVAAVVGSLDASLGRWASRVLLQAG
CR_AGO3    NMPVLGGAGAVPFMVLGADVTHPTGAAARADSRDPSVAAVVASLDASLGRWASRVLLQAG
            ********:*******************************.******************

CR_AGO2    RQEVITGMCGATKELLLEFYRANKQVKPQRLVMYRDGVSEGQFEQVLAEEFTALRRACRE
CR_AGO3    RQEVITGMCGATKELLLEFYRANKQVKPQRLVMYRDGVSEGQFEQVLAEEYTALRRACRE
           **************************************************:*********

CR_AGO2    LEEGYRPAITFVVVQKRHNTRLLPNDRASADPKGNVVPGTVVDSGITAPDGFDFYLNSHS
CR_AGO3    LEEGYRPAITFVVVQKRHNTRLLPSDRAASDQKGNVVPGTVVDSGITAPDGFDFYLNSHA
           ************************.***::* ***************************:

CR_AGO2    GLQGTNKPAHYHVLVDEIGFGADGMQLLTYWLCYLYQRTTKSVSYCPAAYYADRAAFRGR
CR_AGO3    GLQGTNKPAHYHVLVDEIGFGADGIQLLTYWLCYLYQRTTKSVSYCPPAYYADRAAFRGR
           ************************:********************** ************

CR_AGO2    TLLAASSSASDSASESGSRAGRGAGAAEGGASAPPTFAGIHRNLSNVLYFM
CR_AGO3    TLLAASSSASDSASETASRSGRGAGAAEGGASAPPTFAGIHRNLTNVLYFM
           ***************:.**:****************************:******
```

(g)

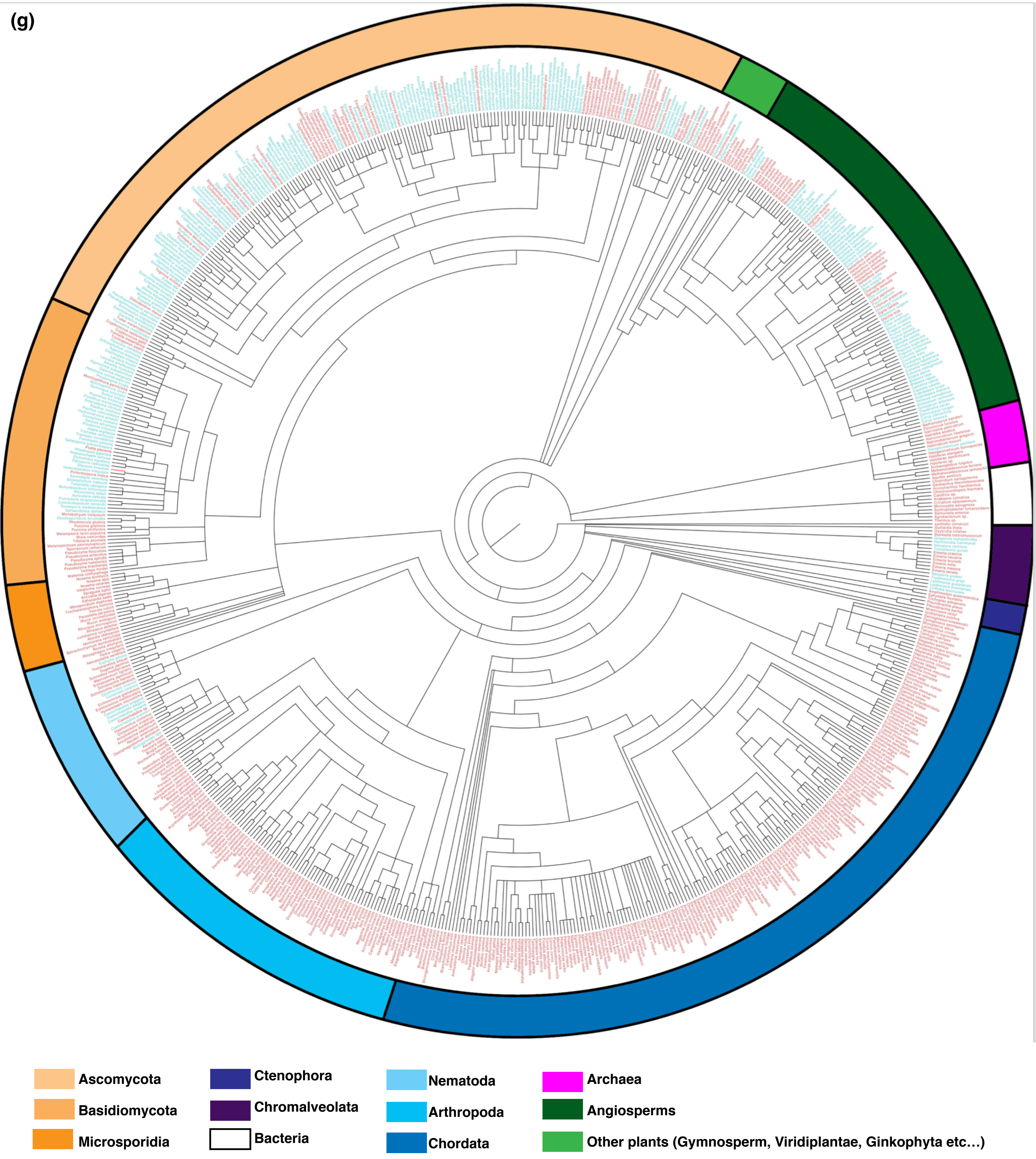

Ascomycota
Basidiomycota
Microsporidia
Ctenophora
Chromalveolata
Bacteria
Nematoda
Arthropoda
Chordata
Archaea
Angiosperms
Other plants (Gymnosperm, Viridiplantae, Ginkophyta etc…)

(h)

>AGO1
MVRKRRTDAPSEGGEGSGSREAGPVSGGGRGSQRGGFQQGGGQHQGGRGYTPQPQQGGRGGRGYGQPPQQQQQYGGPQEYQGRGRGGPPHQGGRGGYGGGRGGGPSSGPPQRQSVPELHQATSPTYQAVSSQPTL
SEVSPTQVPEPTVLAQQFEQLSVEQGAPSQAIQPIPSSSKAFKFPMRPGKGQSGKRCIVKANHFFAELPDKDLHHYDVTITPEVTSRGVNRAVMKQLVDNYRDSHLGSRLPAYDGRKSLYTAGPLPFNSKEFRIN
LLDEEVGAGGQRRERFKVVIKLVARADLHHLGMFLEGKQSDAPQEALQVLDIVLRELPTSRYIPVGRSFYSPDIGKKQSLGDGLESWRGFYQSIRPTQMGLSLNIDMSSTAFIEANPVIQFVCDLLNRDISSRP
LSDADRVKIKKALRGVKVEVTHRGNMRRKYRISGLTAVATRELTFPVDERNTQKSVVEYFHETYGFRIQHTQLPCLQVGNSNRPNYLPMEVCKIVEGQRYSKRLNERQITALLKVTCQRPIDREKDILQTVQLND
YAKDNYAQEFGIKISTSLASVEARILPPPWLKYHESGREGTCLPQVGQWNMMNKKMINGGTVNNWICINFSRQVQDNLARTFCQELAQMCYVSGMAFNPEPVLPPVSARPEQVEKVLKTRYHDATSKLSQGKEID
LLIVILPDNNGSLYGDLKRICETELGIVSQCCLTKHVFKMSKQYMANVALKINVKVGGRNTVLVDALSRRIPLVSDRPTIIFGADVTHPHPGEDSSPSIAAVVASQDWPEITKYAGLVCAQAHRQELIQDLFKEW
KDPQKGVVTGGMIKELLIAFRRSTGHKPLRIIFYRDGVSEGQFYQVLLYELDAIRKACASLEAGYQPPVTFVVVQKRHHTRLFAQNHNDRHSVDRSGNILPGTVVDSKICHPTEFDFYLCSHAGIQGTSRPAHYH
VLWDENNFTADGLQSLTNNLCYTYARCTRSVSIVPPAYYAHLAAFRARFYMEPETSDSGSMASGSMARGGGMAGRSTRGPNVNAAVRPLPALKENVKRVMFYC

>AGO2
MERGGYRGGRGDGRGRGGRGYGGGGGGEQGRDRGYGGGEQGRGRGSERGGGNRGQGRGEQQDFRSQSQRGPPPGHGGRGTTQFQQPRPQVAPQPSQAPASYAGSVGGVAGRGAWGRKPQVPSDSASPSTSTTVV
SEPVRVAEVMNLKPSVQVATSDRKEPMKRPDRGGVVAVRRVNLYVNHYKVNFNPESVIRHYDVEIKGEIPTKKVSRFELAMVRDKVFTDNPDEFPLAMTAYDGQKNIFSAVELPTGSYKVEYPKTEEMRGRSYTF
TIKQVNVLKLGDLKEYMTGRSSFNPRDVLQGMDVVMKEHPSKCMITVGKSFFTRETEPDEDFRFGVIAAKGYRHTLKPTAQGLSLCLDYSVLAFRKAMSVIEYLKLYFNWSDMRQFRRRDVEEELIGLKVTVNHR
KNKQKLTIVGLSMQNTKDIKFDLIDQEGNEPPRKTSIVEYFRIKYGRHIVHKDIPCLDLGKNGRQNFVPMEFCDLVEGQIYPKDNLDKDSALWLKKLSLVNPQQRQRNIDKMIKARNGPSGGEIIGNFGLKVDTN
MTPVEGRVLKAPSLKLAERGRVVREEPNPRQNNQWNLMKKGVTRGSIVKHWAVLDFTASERFNKMPNDFVDNLIDRCWRLGMQMEAPIVYKTSRMETLSNGNAIEELLRSVIDEASRKHGGARPTLVLCAMSRKD
DGYKTLKWIAETKLGLVTQCFLTGPATKGGDQYRANLALKMNAKVGGSNVELMDTFSFFKKEDEVMFIGADVNHPAARDKMSPSIVAVVGTLNWPEANRYAARVIAQPHRKEEIQGFGDACLELVKAHVQATGKR
PNKIVIFRDGVSDAQFDMVLNVELLDVKLTFEKNGYNPKITVIVAQKRHQTRFFPATNNDGSDKGNVPSGTVVDTKVIHPYEYDFYLCSHHGGIGTSKPTHYYTLWDELGFTSDQVQKLIFEMCFTFTRCTKPVS
LVPPVYYADMVAFRGRMYHEASSREKNFKQPRGASTSAASLASSLSSLTIEDKAIFKLHAELENVMFFV

>AGO3
MDRGGYRGGRGDGRGRGGGGDRGRGYSGRGDGRGRGGDGDRGYSGRGDGHGRGGGGDRGRGYSGRGDGRGRGGGGDRGRGYSGRGDGHGRGGGGDRGRGYSGRGRGFVQDRDGGWVNPGQSSGGHVRGRGTQLQQ
PPPQEVPPSSSQAQVSQGVAPGDVGQGGVGDVGRDGVGDVGRDGVGDVGQGGVGDVGQVGVGDVGQGGVGDVGQGGVGDVGRDGVGDVGRDGVGDVGRGGVGDRGQSQSGLSSGHFGRGTQLQQPQPQAVSQSSS
QGQVSQSFATGGVGLGAWARKPQLFSDSTVLPSSSSSNVVASHTASGSQVMTPKPSSSDKKEPVKRPDKGGNIKVKGVINLSVNHFRVSFSTESVIRHYDVDIKGENSSKKISRFELAMVKEKLFKDNNDFPNAM
TAYDGQKNIFSAVELPTGSFKVDFSETEEIMRGRSYTFIIKQVKELKLLDLQAYIDGRSTFIPRDVLQGMDVVMKEHPSKRMITVGKRFFSTRLEIDFGYGVGAAKGFHHTLKPTVQGLSLCLNSSLLAFRKAIS
VIEYLKLYFGWRNIRQFKNCRPDDVVQELIGLKVTVDHRKTKQKFIIMGLSKDDTKDIKFDFIDHAGNQPPRKISIVEYFKEKYGRDIDHKDIPCLNLGKKGRENFVPMEFCNLVEGQIFPKEKLYRDSAAWLKE
LSLVTPQQRLENINKMIKSSDGPRGGDIIGNFGLRVDPNMTTVEGRVLEAPTLKLTDRRGNPIHEKLMSESNQWNLTTKGVTKGSIIKHWAVLDFTASESLKKKMPGYFVNKLIERCKGLGMQMEAPIVCKTSSM
ETLYDGNALEELLRSVIDEASHNHGGACPTLVLCAMTGKHDGYKTLKWIAETKLGLVTQCFLTISAIKGETVSDQYLANLALKINAKVGGTNVELVDNIFSFFKKEDKVMFIGADVNHPAAHDNMSPSIVAVVGT
LNWPEANRYAARVKAQSHRKEEIQGFGETCWELIEAHSQAPEKRPNKIVIFRDGVSDGQFDMVLNVELQNVKDVFAKVGYNPQITVIVAQKRHQTRFFPATTSKDGRAKGNVPSGTVVDTTIIHPFEYDFYLCSQ
HGAIGTSKPTHYYVLSDEIGFNSNQIQKLIFDLCFTFTRCTKPVALVPPVSYADKAASRGRVYYEASLMKKNSKQSRGASSSSASVASSSSSVTMEDKEIFKVHAGIENFMFFV

>AGO4
MDSTNGNGADLESANGANGSGVTEALPPPPPVIPPNVEPVRVKTELAEKKGPVRVPMARKGFGTRGQKIPLLTNHFKVDVANLQGHFFHYSVALFYDDGRPVEQKGVGRKILDKVHQTYHSDLDGKEFAYDGEKT
LFTYGALPSNKMDFSVVLEEVSATRANGNGSPNGNESPSDGDRKRLRRPNRSKNFRVEISYAAKIPLQALANAMRGQESENSQEAIRVLDIILRQHAARQGCLLVRQSFFHNDPTNCEPVGGNILGCRGFHSSFR
TTQGGMSLNMDVTTTMIIKPGPVVDFLIANQNARDPYSIDWSKAKRTLKNLRVKVSPSGQEFKITGLSDKPCREQTFELKKRNPNENGEFETTEVTVADYFRDTRHIDLQYSADLPCINVGKPKRPTYIPLELCA
LVPLQRYTKALTTFQRSALVEKSRQKPQERMTVLSKALKVSNYDAEPLLRSCGISISSNFTQVEGRVLPAPKLKMGCGSETFPRNGRWNFNNKEFVEPTKIQRWVVVNFSARCNVRQVVDDLIKIGGSKGIEIAS
PFQVFEEGNQFRRAPPMIRVENMFKDIQSKLPGVPQFILCVLPDKKNSDLYGPWKKKNLTEFGIVTQCMAPTRQPNDQYLTNLLLKINAKLGGLNSMLSVERTPAFTVISKVPTIILGMDVSHGSPGQSDVPSIA
AVVSSREWPLISKYRASVRTQPSKAEMIESLVKKNGTEDDGIIKELLVDFYTSSNKRKPEHIIIFRDGVSESQFNQVLNIELDQIIEACKLLDANWNPKFLLLVAQKNHHTKFFQPTSPENVPPGTIIDNKICHP
KNNDFYLCAHAGMIGTTRPTHYHVLYDEIGFSADELQELVHSLSYVYQRSTSAISVVAPICYAHLAAAQLGTFMK
FEDQSETSSSHGGITAPGPISVAQLPR
LKDNVANSMFFC

>AGO5
MSNRGGGGHGGASRGRGGGRRSDQRQDQSSGQVAWPGLQQSYGGRGGSVSAGRGRGNVGRGENTGDLTATQVPVASAVSGGRGRGNIGDPTFSVASSSKTVSVASSSKEESKNTEVSETMSNLQITSTETKPEMT
SLPPASSKAVTFPVRPGRGTLGKKVMVRANHFLVQVADRDLYHYDVSINPEVISKTVNRNVMKLLVKNYKDSHLGGKSPAYDGRKSLYTAGPLPFDSKEFVVNLAEKRADGSSGKDRPFKVAVKNVTSTDLYQLQ
QFLDRKQREAPYDTIQVLDVVLRDKPSNDYVSVGRSFFHTSLGKDARDGRGELGDGIEYWRGYFQSLRLTQMGLSLNIDVSARSFYEPIVVTDFISKFLNIRDLNRPLRDSDRLKVKKVLRTLKVKLLHWNGTKS
AKISGISSLPIRELRFTLEDKSEKTVVQYFAEKYNYRVKYQALPAIQTGSDTRPVYLPMELCQIDEGQRYTKRLNEKQVTALLKATCQRPPDRENSIKNLVVKNNYNDDLSKEFGMSVTTQLASIEARVLPPPML
KYHDSGKEKMVNPRLGQWNMIDKKMVNGAKVTSWTCVSFSTRIDRGLPQEFCKQLIGMCVSKGMEFKPQPAIPFISCPPEHIEEALLDIHKRAPGLQLLIVILPDVTGSYGKIKRICETELGIVSQCCQPRQVNK
LNKQYMENVALKINVKTGGRNTVLNDAIRRNIPLITDRPTIIMGADVTHPQPGEDSSPSIAAVVASMDWPEINKYRGLVSAQAHREEIIQDLYKLVQDPQRGLVHSGLIREHFIAFRRATGQIPQRIIFYRDGVS
EGQFSQVLLHEMTAIRKACNSLQENYVPRVTFVIVQKRHHTRLFPEQHGNRDMTDKSGNIQPGTVVDTKICHPNEFDFYLNSHAGIQGTSRPAHYHVLLDENGFTADQLQMLTNNLCYTYARCTKSVSIVPPAYY
AHLAAFRARYYMESEMSDGGSSRSRSSTTGVGQVISQLPAIKDNVKEVMFYC

>AGO6
METSSSLPLSPISIEPEQPSHRDYDITTRRGVGTTGNPIELCTNHFNVSVRQPDVVFYQYTVSITTENGDAVDGTGISRKLMDQLFKTYSSDLDGKRLAYDGEKTLYTVGPLPQNEFDFLVIVEGSFSKRDCGVS
DGGSSSGTCKRSKRSFLPRSYKVQIHYAAEIPLKTVLGTQRGAYTPDKSAQDALRVLDIVLRQQAAERGCLLVRQAFFHSDGHPMKVGGGVIGIRGLHSSFRPTHGGLSLNIDVSTTMILEPGPVIEFLKANQSV
ETPRQIDWIKAAKMLKHMRVKATHRNMEFKIIGLSSKPCNQQLFSMKIKDGEREVPIREITVYDYFKQTYTEPISSAYFPCLDVGKPDRPNYLPLEFCNLVSLQRYTKPLSGRQRVLLVESSRQKPLERIKTLND
AMHTYCYDKDPFLAGCGISIEKEMTQVEGRVLKPPMLKFGKNEDFQPCNGRWNFNNKMLLEPRAIKSWAIVNFSFPCDSSHISRELISCGMRKGIEIDRPFALVEEDPQYKKAGPVERVEKMIATMKLKFPDPPH
FILCILPERKTSDIYGPWKKICLTEEGIHTQCICPIKISDQYLTNVLLKINSKLGGINSLLGIEYSYNIPLINKIPTLILGMDVSHGPPGRADVPSVAAVVGSKCWPLISRYRAAVRTQSPRLEMIDSLFQPIEN
TEKGDNGIMNELFVEFYRTSRARKPKQIIIFRDGVSESQFEQVLKIEVDQIIKAYQRLGESDVPKFTVIVAQKNHHTKLFQAKGPENVPAGTVVDTKIVHPTNYDFYMCAHAGKIGTSRPAHYHVLLDEIGFSPD
DLQNLIHSLSYVNQRSTTATSIVAPVRYAHLAAAQVAQFTKFEGISEDGKVPELPRLHENVEGNMFFC

>AGO7
MEEKTHHHHHSTNKHIPSSKSRTPLLHKPYHHHVQTNPPPFLLHPSSHQNLNLVASNLPSSYYYYYYCYFYSQFHNSLPPPPPPHLLPLSPPLPPLLPLPPPHSMTRFHKSLPVSQVVERKQQHQQKKKIQVSNN
KVSGSIAIEEAALVVAKRPDFGGQDGSVIYLLANHFLVKFDSSQRIYHYNVEISPQPSKEIARMIKQKLVETDRNSFSGVVPAFDGRQNIYSPVEFQGDRLEFFVNLPIPSCKAVMNYGDLREKQPQKKIEKLFR
VNMKLVSKFDGKEQRKEGEDWAPLPPEYIHALDVILRENPMEKCTSIGRSFYSSSMGGSKEIGGGAVGLRGFFQSLRHTQQGLALNMDLSITAFHESIGVIAYLQKRLEFLTDLPRNKGRELSLEEKREVEKALK
NIRVFVCHRETVQRYRVYGLTEEITENIWFPDREGKYLRLMSYFKDHYGYEIQFKNLPCLQISRARPCYLPMELCMICEGQKFLGKLSDDQAAKIMKMGCQKPNERKAIIDKVMTGSVGPSSGNQTREFNLEVSR
EMTLLKGRILQPPKLKLDRPRNLKESKVFKGTRIERWALMSIGGSSDQKSTIPKFINELTQKCEHLGVFLSKNTLSSTFFEPSHILNNISLLESKLKEIQRAASNNLQLIICVMEKKHKGYGDLKRISETRIGVV
TQCCLYPNITKLSSQFVSNLALKINAKIGGSMTELYNSIPSHIPRLLRPDEPVIFMGADVTHPHPFDDCSPSVAAVVGSINWPEANRYVSRMRSQTHRQEIIQDLDLMVKELLDDFYKAVKKLPNRIIFFRDGVS
ETQFKKVLQEELQSIKTACSKFQDYNPSITFAVVQKRHHTRLFRCDPDHENIPPGTVVDTVITHPKEFDFYLCSHLGVKGTSRPTHYHILWDENEFTSDELQRLVYNLCYTFVRCTKPISIVPPAYYAHLAAYRG
RLYIERSSESNGGSMNPSSVSRVGPPKTIPLPKLSDNVKNLMFYC

>AGO9
MDSDEPNGSGLPPPPPFVPANLVPEVEPVKKNILLPMARPRGSGSKGQKIPLLTNHFGVKFNKPSGYFFHYSVAINYEDGRPVEAKGIGRKILDKVQETYQSDLGAKYFAYDGEKTLFTVGALPSNKLDFSVVLE
EIPSSRNHAGNDTNDADRKRSRRPNQTKKFMVEISYAAKIPMQAIASALQGKETENLQDALRVLDIILRQSAARQGCLLVRQSFFHNDVKNFVPIGGGVSGCRGFHSSFRTTQGGLSLNIDTSTTMIVQPGPIVD
FLLANQNKKDPYGMDWNKARRVLKNLRVQITLSNREYKISGLSEHSCKDQLFTWRKPNDKGEFEEVEITVLNYYKERNIEVRYSGDFPCINVGKPKRPTYFPIEFCNLVSLQRYTKSLTNFQRAALVEKSRQKPP
ERMASLTKGLKDSNYNADPVLQDSGVSIITNFTQVEGRILPTPMLKVGKGENLSPIKGKWNFMRKTLAEPTTVTRWAVVNFSARCDTNTLIRDLIKCGREKGINVEPPFKDVINENPQFRNAPATVRVENMFEQI
KSKLPKPPLFLLCILAERKNSDVYGPWKKKNLVDLGIVTQCIAPTRLNDQYLTNVLLKINAKLGGLNSLLAMERSPAMPKVTQVPTIIVGMDVSHGSPGQSDIPSIAAVVSSRQWPLISKYKACVRTQSRKMEMI
DNLFKPVNGKDEGMFRELLLDFYYSSEKRKPEHIIIFRDGVSESQFNQVLNIELDQMMQACKFLDDTWHPKFTVIVAQKNHHTKFFQSRGPDNVPPGTIIDSQICHPRNFDFYLCAHAGMIGTTRPTHYHVLYDE
IGFATDDLQELVHSLSYVYQRSTTAISVVAPVCYAHLAAAQMGTVMKYEELSETSSSHGGITTPGTVPVPPMPQLHNNVSTSMFFC

>AGO10
MPIRQMKDSSETHLVIKTQPLKHHNPKTVQNGKIPPPSPSPVTVTTPATVTQSQASSPSPPSKNRSRRRNRGGRKSDQGDVCMRPSSRPRKPPPPSQTTSSAVSVATAGEIVAVNHQMQMGVRKNSNFAPRPGFG
TLGTKCIVKANHFLADLPTKDLNQYDVTITPEVSSKSVNRAIIAELVRLYKESDLGRRLPAYDGRKSLYTAGELPFTWKEFSVKIVDEDDGIINGPKRERSYKVAIKFVARANMHHLGEFLAGKRADCPQEAVQI
LDIVLRELSVKRFCPVGRSFFSPDIKTPQRLGEGLESWCGFYQSIRPTQMGLSLNIDMASAAFIEPLPVIEFVAQLLGKDVLSKPLSDSDRVKIKKGLRGVKVEVTHRANVRRKYRVAGLTTQPTRELMFPVDEN
CTMKSVIEYFQEMYGFTIQHTHLPCLQVGNQKKASYLPMEACKIVEGQRYTKRLNEKQITALLKVTCQRPRDRENDILRTVQHNAYDQDPYAKEFGMNISEKLASVEARILPAPWLKYHENGKEKDCLPQVGQWN
MMNKKMINGMTVSRWACVNFSRSVQENVARGFCNELGQMCEVSGMEFNPEPVIPIYSARPDQVEKALKHVYHTSMNKTKGKELELLLAILPDNNGSLYGDLKRICETELGLISQCCLTKHVFKISKQYLANVSLK
INVKMGGRNTVLVDAISCRIPLVSDIPTIIFGADVTHPENGEESSPSIAAVVASQDWPEVTKYAGLVCAQAHRQELIQDLYKTWQDPVRGTVSGGMIRDLLISFRKATGQKPLRIIFYRDGVSEGQFYQVLLYEL
DAIRKACASLEPNYQPPVTFIVVQKRHHTRLFANNHRDKNSTDRSGNILPGTVVDTKICHPTEFDFYLCSHAGIQGTSRPAHYHVLWDENNFTADGIQSLTNNLCYTYARCTRSVSIVPPAYYAHLAAFRARFYL
EPEIMQDNGSPGKKNTKTTTVGDVGVKPLPALKENVKRVMFYC

(i)

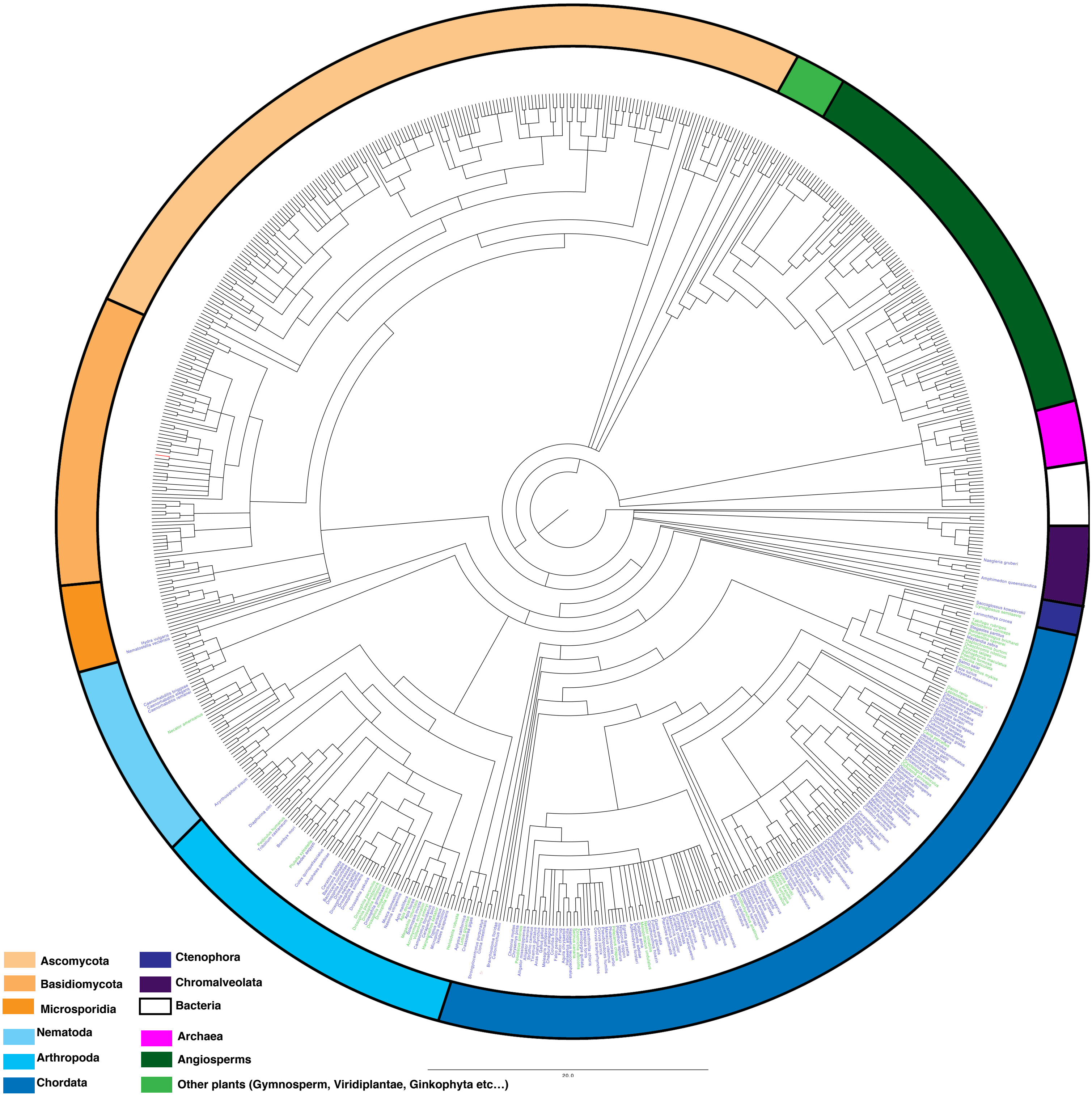
Ascomycota
Basidiomycota
Microsporidia
Nematoda
Arthropoda
Chordata
Ctenophora
Chromalveolata
Bacteria
Archaea
Angiosperms
Other plants (Gymnosperm, Viridiplantae, Ginkophyta etc…)
20.0

(j)

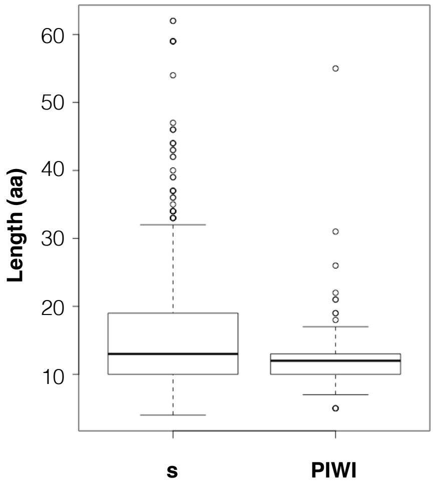
60
50
40
30
20
10
Length (aa)
s
PIWI

Supplementary Figure 2

(a)

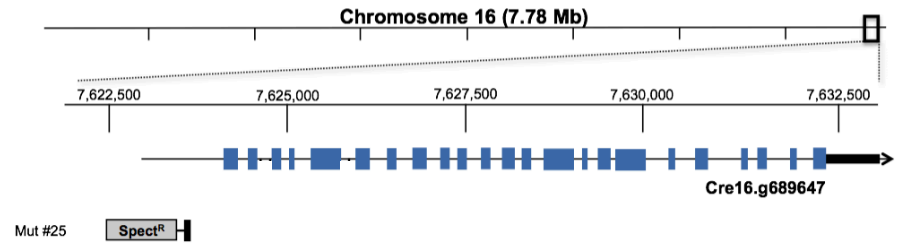

(b)

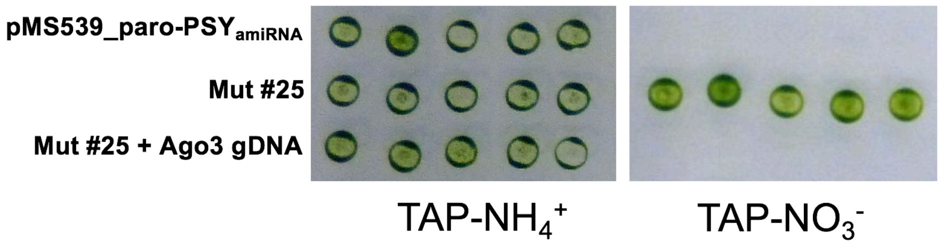

(c)

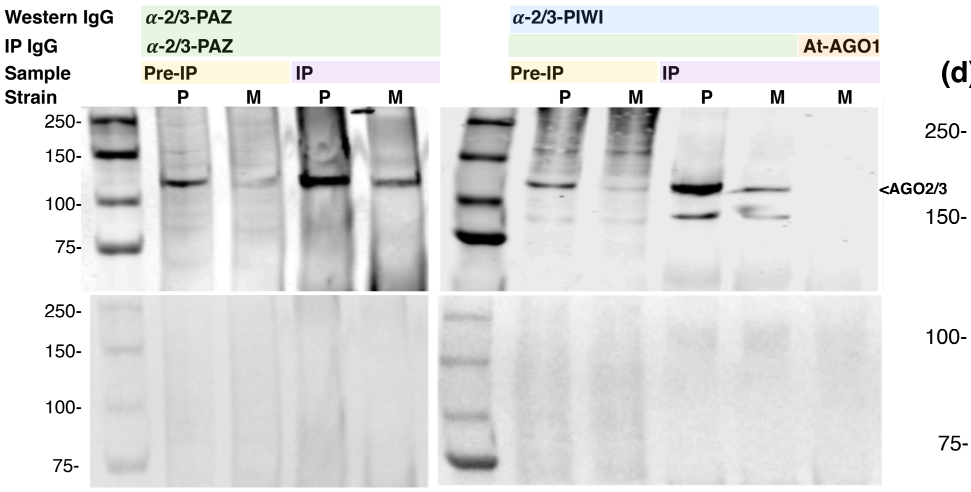

(d)

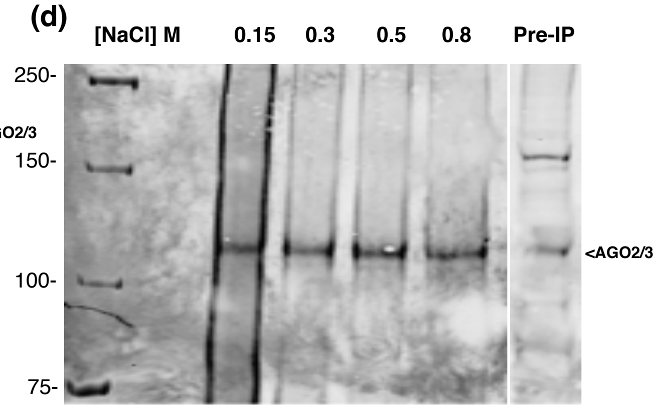

(e)

```
   1 MSGRGRGGGG GYGGGYGGGG GGSRGGGGGG GSGGGDRGYG GGGGGRGGGG
  51 YGGGSAGGGG YGGGGGRGGG GGGGGYGGGG GGRGGGGGGG GVVSPQQAAA
 101 TLDVVLKSAR TLTAKVNVET TPDGRPLGIT RRPNAGTVGK AVALLANYFA
 151 LATTPAFPGQ AYHYDVEIRS VEEAAGGGGG GGRGGGRGGG GGGGGGRGGR
 201 RDLAPASATG DVPGGAAAAA AAEDLPPRLA HRVMAAAAAA HGWPAGAWRF
 251 DGRKNLFLPG ELLPREVREW PVTLKPREGD KSERDKGFVV ATKWAACVGL
 301 TQLQDYLAQR QQTAPRDAMQ VLDIVIRHAF AIDPRCTVVG RGFYYGGEGV
 351 MPLGGGAEVW SGFQQSFKAV QAGLMLNLDS SFAAFMSARP LPELLAEGAG
 401 VRGGPAQLAA ADPRRLRAAA RSLVGFKVEF PMPGGRARRK MLTGLSEQGA
 451 DRTMFMNEKE GREMSVAEYF RSTGRPLRHP GLPCANVGDR RRAVYIPLEL
 501 CTVVAGQRRM KLDATQSAGM ITAAKQDPAV KAEACNKQAK RVAEALAAGG
 551 TERSWGLKLG TGMLPVQGRV LPNPVLQYGN RQDFDAGPLG SWNTLNVKFV
 601 DARALDSWAV AVMMNQADVD FDGDNSIWKF LEDLCSAMIT RGMRVASPVT
 651 RGSNDSPPVE YGGAAAGGGA AGRGGGARGV EATLRAAADA AAARYKKPAQ
 701 LVLVVLPVKP SDEYREVKRV SDIELGIPSQ VVVGGKARVG YREQRGGGPQ
 751 YCANVAMKIN NKLGGVNVQL SGGLRYMPVL GGAGSVPFMV LGADVTHPTG
 801 AAARADSRDP SVAAVVGSLD ASLGRWASRV LLQAGRQEVI TGMCGATKEL
 851 LLEFYRANKQ VKPQRLVMYR DGVSEGQFEQ VLAEEFTALR RACRELEEGY
 901 RPAITFVVVQ KRHNTRLLPN DRASADPKGN VVPGTVVDSG ITAPDGFDFY
 951 LNSHSGLQGT NKPAHYHVLV DEIGFGADGM QLLTYWLCYL YQRTTKSVSY
1001 CPAAYYADRA AFRGRTLLAA SSSASDSASE SGSRAGRGAG AAEGGASAPP
1051 TFAGIHRNLS NVLYFM
```

(f)

```
MSGRGRGGGG GYGGGGGYGG GGGGGRGGGG GDRGGGGYGG GGRGGGGGGG
YGGGGGGGGR GGGGGGGYGG GGGRGGGGGG GGGGVVSPAQ AAASLDAILK
SAKSLTAPVK VETTADGRPL GITRRPSAGS VGKAVALLAN YFALATTPAF
PGQAYHYDVE IRSVETPGGG GGGGRGGGRG GGRGGGRGGR GGAAPAPATA
EESAGAPAAE AGGEDLPPRL AHRVMASAAA AHGWPAGAWR FDGRKNLFLP
GELLPREVRE WPVTLKPREG DKSERDKGFV VTTKWAACVG LTQLQDYLAR
RQQTAPRDAM QVLDIVIRHA FAIDPRCTVV GRGFYYGGEG VMPLGGGAEV
WSGFQQSFKA VQAGLTLNLD SSFAAFMSAR PLPELLAEGA GVRGGPAQLA
AADPHRLRAA ARGLAGFKVE FPMPGGRARR KALVGLSEQG ADRTMFMNEK
EGREMSVAEY FRSTGRPLRH PGLPCANVGD RRRAVFIPVE LCTVVAGQRR
MKLDATQSAG MITAAKQDPA VKKEACDKQA KRVAEALAAG GTDRCWGLKL
ATGMLPVQGR VLPNPVLQYG DPQAFDAGPL GSWNLRNVKF VDPRALDSWG
VAVMMNQADV DFDGDNSLWQ FLEDLTGDMI RCGMRVASPV TAASTDSPPV
EFGGMPGGGG RGGGRGIEAT MRAAADAAAA RYKKPAQLVL VVLPEKTADE
YREVKRVSDI ELGIPSQVVV ASKARVGYRA HKGGGPQYCA NVAMKINNKL
GGVNVQLSGG LRNMPVLGGA GAVPFMVLGA DVTHPTGAAA RADSRDPSVA
AVVASLDASL GRWASRVLLQ AGRQEVITGM CGATKELLLE FYRANKQVKP
QRLVMYRDGV SEGQFEQVLA EEYTALRRAC RELEEGYRPA ITFVVVQKRH
NTRLLPSDRA ASDQKGNVVP GTVVDSGITA PDGFDFYLNS HAGLQGTNKP
AHYHVLVDEI GFGADGIQLL TYWLCYLYQR TTKSVSYCPP AYYADRAAFR
GRTLLAASSS ASDSASETAS RSGRGAGAAE GGASAPPTFA GIHRNLTNVL
YFM
```

(g)

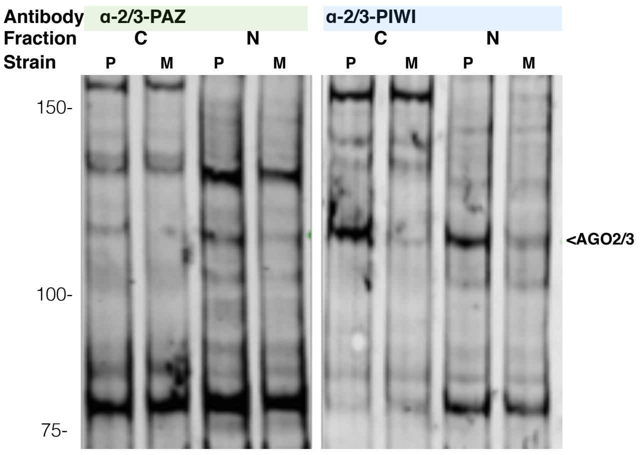

(h)

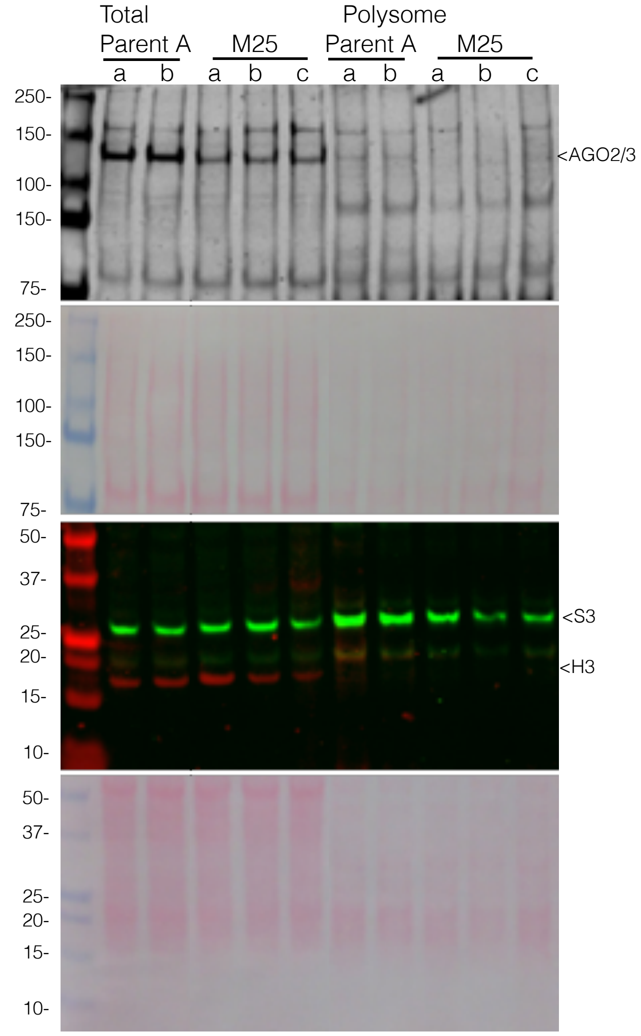

Total
Polysome
Parent A
M25
Parent A
M25
a b a b c a b a b c
250-
150-
100-
150-
75-
250-
150-
100-
150-
75-
50-
37-
25-
20-
15-
10-
50-
37-
25-
20-
15-
10-
<AGO2/3
<S3
<H3

(i)

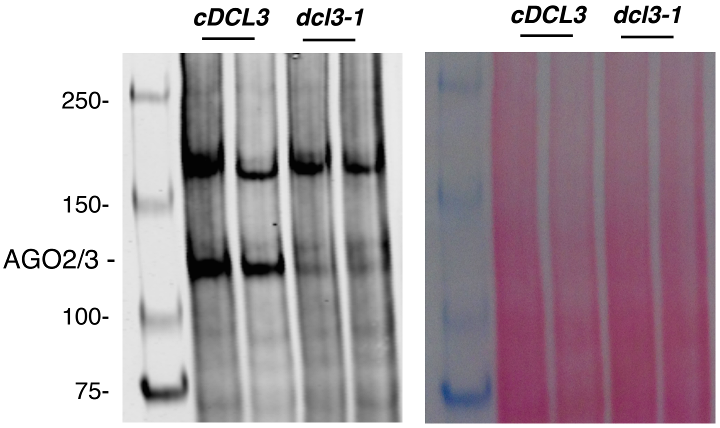

cDCL3
dcl3-1
250-
150-
AGO2/3 -
100-
75-
cDCL3
dcl3-1

(j)

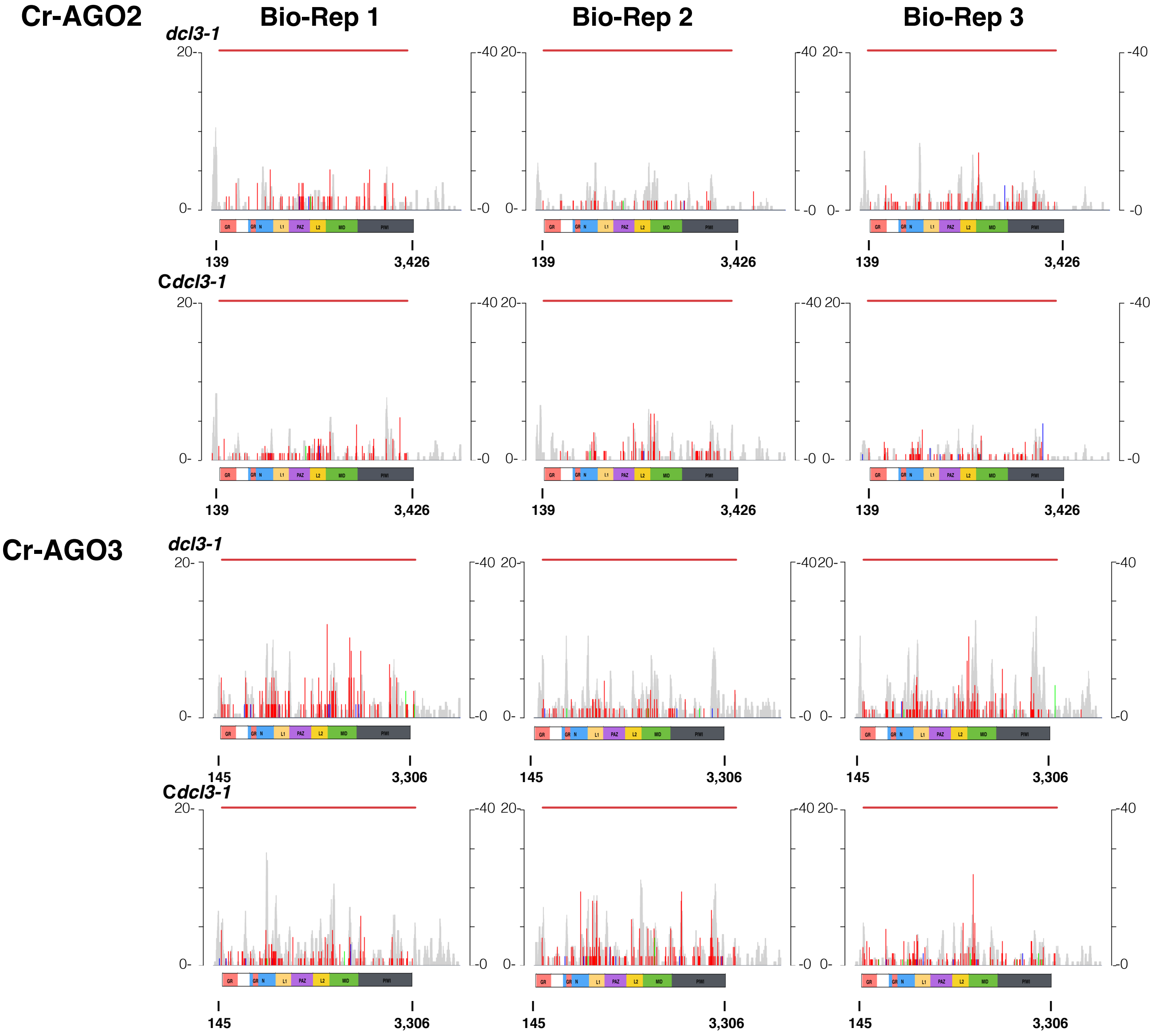

Cr-AGO2
Bio-Rep 1
Bio-Rep 2
Bio-Rep 3
dcl3-1
Cdcl3-1
20-
0-
40
0
139
3,426
Cr-AGO3
145
3,306

Supplementary Figure 3:

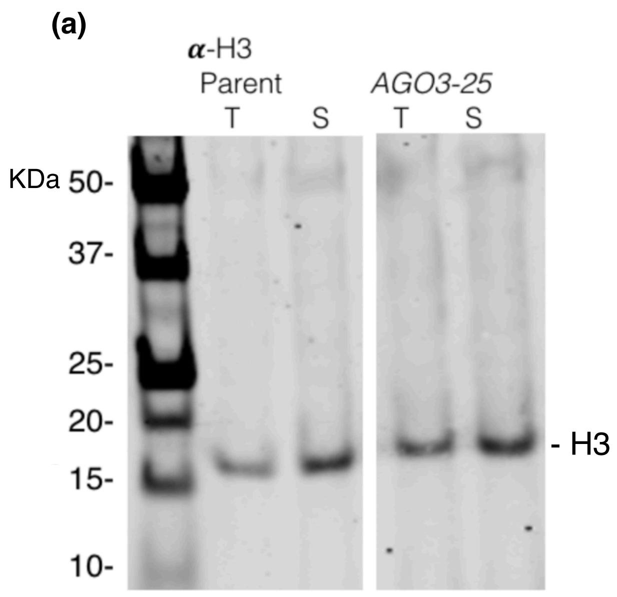

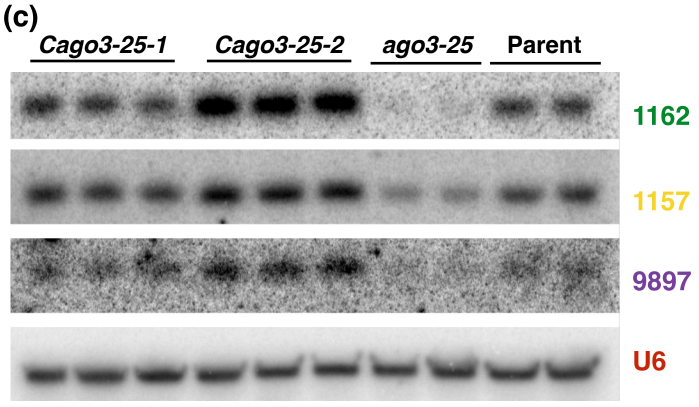

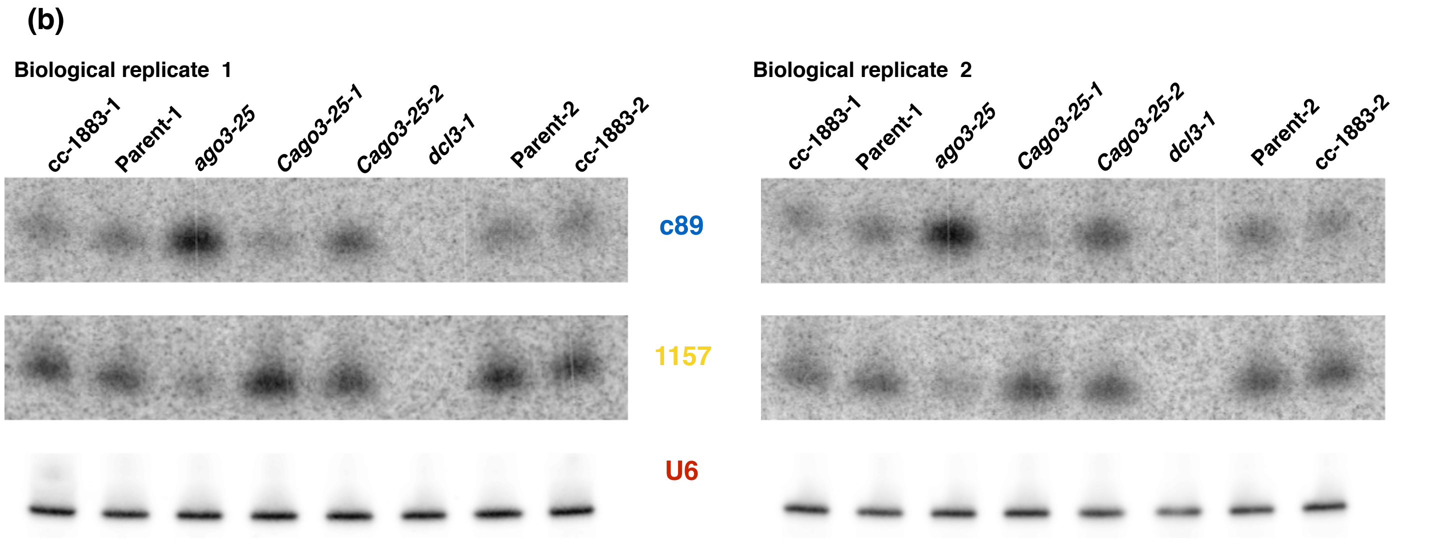

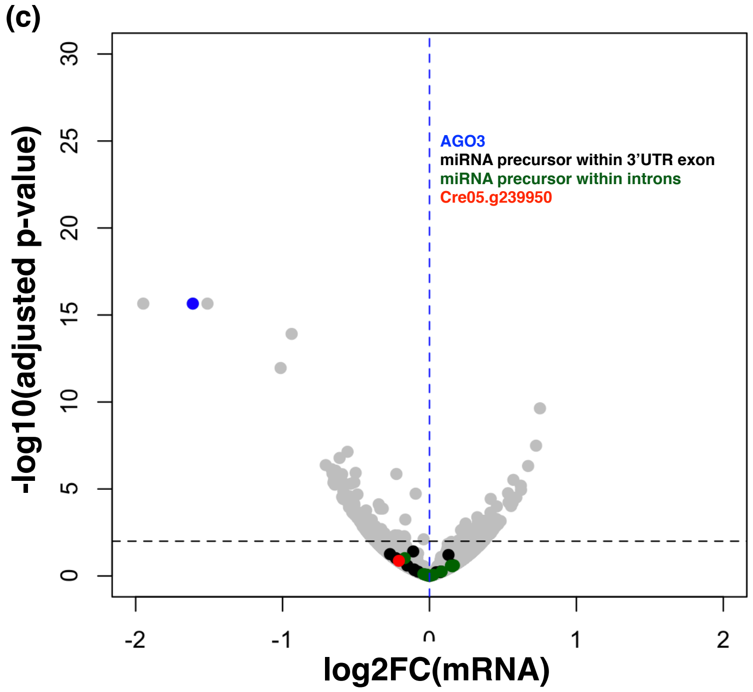

Supplementary table 1: Normalised siRNA abundance

	Parent-1	Parent-2	Parent-3	AGO3-25-1	AGO3-25-2	AGO-3-25-3
**chromosome_4_741459_742332**	0.225254615	0.385155893	0.357669483	0.113589486	0.091178661	0.058826061
**chromosome_16_6192827_6193080**	0.08787003	0.050387576	0.049798413	0.069023079	0.059133533	0.061418595
**chromosome_13_4823025_4823624**	0.088036272	0.058830454	0.056438984	0.02991756	0.059852016	0.115443414
**chromosome_7_2881984_2882520**	0.07019275	0.043011013	0.03956098	0.028001714	0.012474047	0.02965377
**chromosome_17_3465102_3465976**	0.033949784	0.026081158	0.021870839	0.024462116	0.024714487	0.02570224
**chromosome_8_347555_348048**	0.008299288	0.014461389	0.016135801	0.023423156	0.079165775	0.022127078
**chromosome_1_2125447_2125697**	0.026821677	0.018962975	0.02513608	0.022597673	0.018240576	0.028326648
**chromosome_17_5152761_5152938**	0.020906978	0.02243839	0.017035468	0.017913888	0.015943854	0.0186455
**chromosome_5_910267_910743**	0.032750808	0.024551229	0.022863	0.014792777	0.01049267	0.01616418
**chromosome_13_791487_791911**	0.030753227	0.026548165	0.021174207	0.014154766	0.018693664	0.017470281
**chromosome_12_6166769_6167265**	0.017742493	0.014539977	0.01194653	0.013537317	0.014355523	0.017041695
**chromosome_14_1191493_1191841**	0.016124971	0.012332719	0.009841742	0.012331446	0.010614267	0.01776595
**chromosome_3_6440818_6442758**	0.017448496	0.012697391	0.010178779	0.010953827	0.010073892	0.014071436
**chromosome_13_2348229_2348355**	0.017415889	0.012691172	0.010940834	0.010699832	0.010854939	0.012806702
**scaffold_21_122657_123081**	0.02680564	0.019513094	0.017529969	0.00992031	0.019663413	0.013061683
**chromosome_3_8398694_8400260**	0.001768262	0.00251087	0.002894816	0.009542946	0.002299245	0.004003747
**chromosome_11_2205569_2205928**	0.008104714	0.005369451	0.005207601	0.008776124	0.00949517	0.012134664
**chromosome_15_112650_114047**	0.023366402	0.011217216	0.010259542	0.008407832	0.006647982	0.008756163
**chromosome_17_195871_195937**	0.016894711	0.018972021	0.015232976	0.00778494	0.009807993	0.010142284
**chromosome_17_194526_194857**	0.014641619	0.010025387	0.007875921	0.007564811	0.00809756	0.009877809
**chromosome_3_6838785_6839658**	0.00727083	0.012318584	0.010044325	0.007498893	0.018547848	0.007391742
**chromosome_11_1337240_1337592**	0.006121566	0.004089422	0.004011504	0.00673449	0.005540489	0.009338008
**chromosome_9_893836_894970**	0.00748037	0.006768776	0.006121254	0.006491985	0.00568025	0.007174737
**chromosome_4_619389_619451**	0.003793639	0.001856155	0.002986407	0.006442396	0.004068205	0.008076665
**chromosome_5_935248_935504**	0.012154399	0.00962453	0.008412833	0.006355312	0.005388114	0.008634097
**scaffold_21_46903_47707**	0.006196402	0.010474302	0.008220627	0.005608446	0.013351968	0.005642815
**chromosome_8_448814_450085**	0.007377204	0.004277694	0.003543623	0.005486287	0.004015732	0.005905934
**chromosome_3_4522839_4523210**	0.008409403	0.006799872	0.005589305	0.005288534	0.00480132	0.006388093
**chromosome_4_3389815_3390660**	0.00339113	0.003251523	0.002389938	0.004616657	0.010065314	0.006801081
**chromosome_17_7004995_7005721**	0.004782006	0.004313314	0.003764253	0.003915148	0.003837625	0.004635775
**chromosome_4_457766_457962**	0.00294853	0.001291902	0.001608933	0.003626683	0.003470815	0.004390966
**chromosome_11_427007_427373**	0.007438676	0.005614827	0.005307764	0.003595236	0.005403251	0.00326932
**chromosome_1_1695652_1695774**	0.008485842	0.00678913	0.005294228	0.003545646	0.006720637	0.003355444
**chromosome_5_2294486_2295191**	0.000126686	0.000149827	7.35435E-05	0.00351178	0.012746001	0.000111893
**chromosome_11_1857326_1857429**	0.008978689	0.008433266	0.006478143	0.003376921	0.00310451	0.003328318
**chromosome_7_3850572_3850929**	0.009181815	0.007827174	0.006020188	0.003372688	0.003210466	0.003347306
**chromosome_13_2301408_2301719**	0.013716863	0.017046324	0.014358576	0.002851394	0.003075751	0.006004943
**chromosome_14_1950078_1950361**	0.006959193	0.007002845	0.005650215	0.002823575	0.004836639	0.004779541
**chromosome_2_9207643_9207901**	0.002606423	0.004505544	0.00310552	0.00237727	0.001410728	0.002451481
**chromosome_11_422391_422757**	0.003780276	0.002453201	0.002446787	0.001777359	0.001704882	0.001620758
**scaffold_19_167877_168751**	0.000545232	0.001031826	0.000887485	0.001724141	0.004582344	0.001501405
**chromosome_11_433372_433738**	0.002969377	0.001970363	0.002100276	0.001696927	0.001436965	0.00150683
**chromosome_10_5038594_5038755**	0.001537341	0.001362575	0.001823698	0.001608029	0.006842234	0.001870992
**chromosome_6_4030753_4031503**	0.00343122	0.002458289	0.001875584	0.001601377	0.001643327	0.002151743
**chromosome_6_4087982_4088721**	0.003264978	0.002386485	0.001865207	0.001516712	0.001578744	0.002125295
**chromosome_5_2986270_2986667**	0.003025503	0.002513131	0.002249167	0.001504617	0.001889548	0.001750961
**chromosome_10_778202_778490**	5.55923E-05	4.63615E-05	3.02295E-05	0.001465913	0.004545512	4.54355E-05
**chromosome_9_6365908_6366037**	0.002865141	0.002125278	0.002013648	0.001396971	0.002416301	0.001915072
**chromosome_3_6582826_6583191**	0.00214458	0.001791702	0.001581862	0.001387295	0.000898608	0.001310169
**chromosome_2_476786_476830**	0.001816906	0.002244574	0.001449213	0.001314725	0.001649382	0.001514968
**chromosome_7_4783768_4794684**	0.000559665	0.000977549	0.000752129	0.001269369	0.001051487	0.003404948
**chromosome_10_2431974_2432405**	0.002729902	0.002094182	0.001729851	0.001250017	0.001241199	0.001542772
**chromosome_16_1558354_1558399**	0.001331542	0.00091253	0.000879363	0.001150233	0.000682155	0.001087061
**chromosome_9_3000600_3006834**	0.00036723	0.000539942	0.000413287	0.001048031	0.000804761	0.002389092
**chromosome_10_2432800_2433146**	0.00153253	0.001083841	0.001013817	0.000961552	0.000810311	0.001219298
**chromosome_16_7701450_7707096**	0.000504607	0.000919314	0.000628504	0.000940385	0.000748756	0.002903124
**chromosome_8_186488_192134**	0.000470396	0.000892176	0.000630308	0.000931919	0.000784074	0.002899055
**chromosome_4_2915643_2921289**	0.00049552	0.000842422	0.000624894	0.000910148	0.000782056	0.002789196
**chromosome_9_6156833_6158503**	0.001972992	0.001355225	0.001264226	0.000908334	0.001043919	0.001415959
**chromosome_6_2556368_2562014**	0.000489105	0.000875214	0.000606847	0.000891401	0.000786093	0.002805471
**chromosome_1_6561109_6566755**	0.000482691	0.000885957	0.000620833	0.000884748	0.000800725	0.00290448
**chromosome_4_585019_585290**	0.000656417	0.000408773	0.000543229	0.000878096	0.000768433	0.001137921
**chromosome_9_6518825_6519075**	0.005775184	0.004490279	0.003245839	0.00086842	0.001334541	0.001150806
**chromosome_7_4352836_4361019**	0.000359746	0.00061118	0.000465174	0.000829112	0.000645827	0.002333485
**chromosome_2_1328804_1334369**	0.000458636	0.000793799	0.000566691	0.000823064	0.000668532	0.00266713
**chromosome_9_5020215_5020525**	0.00040732	0.001520883	0.002842478	0.000814598	0.001851203	0.001280331
**chromosome_1_3243522_3249183**	0.000440997	0.000830549	0.000580227	0.000803107	0.000713437	0.002494204
**chromosome_7_860854_865923**	0.000452222	0.000775141	0.000571654	0.00079706	0.000684173	0.002704428
**chromosome_1_1124614_1125494**	0.00182332	0.002329947	0.001468614	0.000790408	0.001632731	0.002151743

Supplementary table 1: cont.

	Parent-1	Parent-2	Parent-3	AGO3-25-1	AGO3-25-2	AGO-3-25-3
**chromosome_11_1367777_1373430**	0.000437255	0.000800018	0.000579324	0.000762589	0.000742196	0.002149708
**chromosome_7_4518519_4524172**	0.000464516	0.000783622	0.000571203	0.000737794	0.000711923	0.002189041
**chromosome_12_2358008_2363661**	0.000438858	0.000726518	0.000594664	0.000728723	0.000734628	0.002144283
**chromosome_4_3690316_3690707**	0.000930636	0.00090631	0.000787773	0.000726304	0.000477811	0.000725611
**chromosome_15_109180_110407**	0.002479202	0.001192395	0.000936664	0.000717838	0.000973786	0.000994155
**chromosome_5_1883558_1889064**	0.000380059	0.000661499	0.000499013	0.000699695	0.000567621	0.002093423
**chromosome_2_8282593_8282661**	0.000929033	0.000785318	0.00066505	0.000698486	0.000700823	0.000726968
**chromosome_12_6402227_6402312**	0.00111238	0.00088426	0.000717387	0.000679738	0.001057542	0.000752059
**chromosome_9_6821854_6827511**	0.000289721	0.000461353	0.000397947	0.000675505	0.000492443	0.001980173
**scaffold_42_5629_10706**	0.000393422	0.000683549	0.000488636	0.000668248	0.000669541	0.002399265
**chromosome_6_6776067_6776481**	0.000957898	0.000915922	0.000729118	0.000667643	0.000730592	0.000825976
**chromosome_16_6024629_6029942**	0.00029079	0.000470399	0.000360498	0.000656153	0.000479325	0.002028999
**chromosome_3_4443661_4449329**	0.000273685	0.00048623	0.000333427	0.000647082	0.000505561	0.001951691
**chromosome_6_4364533_4370186**	0.000323397	0.000544465	0.000397045	0.000642849	0.000599913	0.001616011
**chromosome_6_4989009_4994432**	0.000245889	0.000429692	0.000320794	0.00064043	0.000433915	0.001877096
**chromosome_1_6258205_6264270**	0.000284376	0.000460222	0.000342451	0.00063922	0.000487902	0.002009333
**chromosome_12_9137554_9143184**	0.000271547	0.000477749	0.000347414	0.000631963	0.000510102	0.002014758
**chromosome_10_1135214_1140860**	0.000273151	0.000472096	0.000363657	0.000631358	0.000488407	0.002022896
**chromosome_9_7026197_7031843**	0.000265132	0.000451742	0.000377192	0.000628335	0.000478316	0.002037815
**chromosome_2_8969911_8975557**	0.000280634	0.000491319	0.00034967	0.00062652	0.000496984	0.002009333
**chromosome_7_74484_75443**	0.001097413	0.000735564	0.000618577	0.000623497	0.000709905	0.000556754
**chromosome_1_6436501_6442155**	0.00029079	0.000438738	0.000311319	0.000588421	0.000454097	0.001751639
**chromosome_5_1111605_1117062**	0.000307896	0.000500365	0.000379448	0.000584188	0.000533816	0.001234217
**chromosome_12_2335855_2340967**	0.000231456	0.00042743	0.000300039	0.000581164	0.000433915	0.001776731
**chromosome_10_5335959_5336343**	0.000684213	0.000529765	0.000404264	0.000571488	0.001113042	0.000588627
**chromosome_11_3085875_3090959**	0.000281169	0.000457961	0.000390277	0.000569674	0.000533816	0.001578035
**chromosome_16_580543_580860**	0.000389146	0.000436476	0.000397496	0.000567255	0.001322432	0.000658475
**chromosome_5_1535968_1541622**	0.000257649	0.000450046	0.000319892	0.000562417	0.000461161	0.001807925
**chromosome_13_5036438_5036905**	0.001258845	0.001012603	0.001112627	0.000558184	0.000488407	0.000918204
**chromosome_10_5682660_5683283**	0.001375375	0.001087233	0.001175793	0.000553951	0.000540376	0.000991443
**chromosome_4_667365_672440**	0.000199384	0.000320573	0.000254018	0.000546694	0.000450061	0.000968386
**chromosome_7_75746_77085**	0.000807692	0.00055238	0.000457504	0.000536413	0.00061959	0.000515388
**chromosome_2_4103879_4109497**	0.000288118	0.000452307	0.000371778	0.000533389	0.000495975	0.001209804
**chromosome_1_6965122_6965957**	0.001178663	0.000896699	0.000773335	0.000527946	0.000645322	0.000844964
**chromosome_2_3660917_3665372**	0.000307361	0.000566515	0.000413738	0.000525527	0.000487902	0.001808603
**chromosome_3_6909023_6909784**	0.001148729	0.000881433	0.000739044	0.000503756	0.000591335	0.000783253
**chromosome_2_3688285_3688612**	0.000343175	0.000358453	0.00037629	0.000492871	0.000358737	0.000720186
**chromosome_7_1805184_1806219**	0.001069617	0.000868995	0.00071468	0.000491661	0.000505057	0.000661866
**chromosome_14_3593890_3596553**	0.000310568	0.000394638	0.000332525	0.000483195	0.000452583	0.000754093
**chromosome_12_6158446_6158635**	0.000817314	0.000837334	0.000591055	0.000423325	0.000520698	0.00047063
**chromosome_11_448833_449199**	0.000625413	0.00046418	0.000395691	0.000406392	0.000809302	0.000357381
**chromosome_12_6177015_6177204**	0.000817314	0.000870691	0.000549997	0.000405787	0.000504048	0.000458424
**chromosome_11_442682_443048**	0.000595479	0.000481142	0.000397496	0.000400344	0.000767929	0.000400782
**chromosome_14_187995_188469**	0.000859008	0.000654149	0.000602786	0.000394297	0.000449052	0.000608293
**chromosome_14_189484_190319**	0.000624879	0.000424603	0.000390728	0.000391878	0.000353187	0.000516744
**chromosome_12_6184630_6184819**	0.000694904	0.000369761	0.000351475	0.000390063	0.000409696	0.000487584
**chromosome_17_1036775_1037166**	0.000562872	0.000584607	0.000476002	0.000384016	0.00024824	0.000366196
**chromosome_3_7137470_7137553**	0.000678867	0.000550119	0.00039524	0.000377364	0.000514139	0.000404172
**chromosome_17_2281456_2281644**	0.000672453	0.000590826	0.000457955	0.000377364	0.000596885	0.000351955
**chromosome_3_6910803_6911638**	0.000607773	0.000451742	0.00041058	0.000376154	0.000361259	0.000562179
**chromosome_5_2985467_2985777**	0.000561268	0.000452307	0.000438554	0.000344102	0.000527762	0.00042316
**chromosome_12_3262538_3263099**	0.000745685	0.000660934	0.000715131	0.000328379	0.000312822	0.000606937
**chromosome_1_6966974_6967735**	0.000578374	0.000415557	0.000369522	0.00032475	0.00032695	0.000497078
**chromosome_17_1966528_1966835**	0.00047788	0.000390115	0.000362754	0.000273347	0.00022503	0.000311945
**chromosome_3_6916915_6917412**	0.000378455	0.000311527	0.000271163	0.000244319	0.000228562	0.000343818
**chromosome_7_5559507_5559571**	0.000160362	0.000123819	0.000115053	0.00024069	0.000108479	0.000153938
**chromosome_3_3760235_3760258**	4.59705E-05	6.78461E-05	6.58733E-05	0.000234038	0.000277504	0.000178351
**chromosome_12_8402766_8403646**	0.000540421	0.000299088	0.000253567	0.000219524	0.00026489	0.000322117
**chromosome_9_7770371_7770651**	0.000205264	0.000200711	0.000165586	0.000214081	0.000134211	0.000217005
**chromosome_8_436979_438264**	0.001158351	0.000445522	0.00044487	0.000208034	0.00015742	0.00022989
**chromosome_12_3577642_3577772**	0.000117599	0.000195623	0.000152501	0.00020622	0.000361764	0.000354668
**chromosome_5_3227660_3227758**	0.000110115	0.000139084	0.000138063	0.000198358	0.000250763	0.000292279
**chromosome_7_1807237_1808012**	0.000365092	0.000231807	0.000227398	0.000191706	0.000199298	0.000298382
**chromosome_7_692621_693635**	0.000197246	0.000163396	0.000151599	0.000189287	0.000150861	0.000271935
**chromosome_11_1347622_1350738**	6.30759E-05	0.0001617	0.000119113	0.00018203	0.000159943	0.001006362
**chromosome_2_9173275_9173436**	0.000297739	0.000277038	0.000225142	0.000165097	0.000149852	0.000190558
**chromosome_10_3237925_3237980**	0.000219696	0.000339796	0.00017867	0.00014514	0.000217967	0.000172926
**chromosome_4_591752_592023**	0.000233595	0.000240288	0.000175512	0.000133045	0.000320391	0.000229212

Supplementary table 1: cont.

	Parent-1	Parent-2	Parent-3	AGO3-25-1	AGO3-25-2	AGO-3-25-3
chromosome_7_3068787_3069348	7.26976E-05	9.78114E-05	7.26411E-05	0.000131835	0.000105451	0.000159363
chromosome_17_4288631_4288910	0.000187624	0.000145304	0.000177768	0.000121555	7.97193E-05	0.000203442
chromosome_2_8349154_8349307	0.000167311	0.000160004	0.000189047	0.00011974	7.87102E-05	0.000221752
chromosome_10_3399871_3400004	0.000179071	0.000201277	0.000147989	0.000118531	0.000256313	0.000126134
chromosome_12_9155586_9156147	5.77305E-05	9.95076E-05	0.000084372	0.000114902	0.000105956	0.000168179
chromosome_3_7129085_7129168	0.000104236	8.81999E-05	7.08364E-05	0.000113088	7.11419E-05	9.76524E-05
chromosome_9_1108955_1109026	0.000218627	0.000219934	0.000110541	0.000106436	9.18285E-05	0.000160041
chromosome_4_572391_572455	0.000120806	8.19807E-05	9.79076E-05	9.49457E-05	0.000172557	0.00014241
chromosome_16_4565554_4566037	0.000279565	0.000152088	0.000153404	8.46649E-05	8.77921E-05	0.000105112
chromosome_13_2306295_2307879	0.000019778	5.25807E-05	3.47414E-05	8.46649E-05	5.95372E-05	0.000148513
chromosome_1_3120583_3120625	0.000154482	6.44538E-05	9.47493E-05	7.37794E-05	5.19689E-05	8.34114E-05
chromosome_12_6167927_6169267	0.000127221	8.31114E-05	7.26411E-05	7.19652E-05	9.93968E-05	0.000100365
chromosome_2_403293_403346	0.000159828	0.000121558	9.20422E-05	6.83367E-05	5.85281E-05	6.91704E-05
chromosome_10_4584909_4585470	2.24507E-05	2.99653E-05	1.98522E-05	5.44275E-05	5.70144E-05	6.30672E-05
chromosome_2_9129503_9129616	0.000626482	0.000226719	0.000192657	0.00004838	4.64188E-05	2.91601E-05
chromosome_14_1912136_1912559	7.69739E-05	0.000048623	0.000041058	4.47515E-05	6.81146E-05	8.88365E-05
chromosome_12_3078920_3079187	0.000032607	3.90115E-05	2.30105E-05	3.74945E-05	3.43096E-05	3.45852E-05
chromosome_9_7449955_7450222	4.32978E-05	4.24038E-05	3.33878E-05	3.50755E-05	3.27959E-05	4.06885E-05
chromosome_1_8028918_8029209	3.63488E-05	5.99307E-05	3.15831E-05	0.000033866	4.03642E-05	3.05164E-05
chromosome_4_588926_588995	9.24756E-05	4.07076E-05	5.00818E-05	3.26565E-05	4.03642E-05	6.78141E-05
chromosome_13_3152367_3152569	0.000112788	0.000123819	8.84327E-05	3.08422E-05	3.73369E-05	0.000057642
chromosome_10_6414325_6414592	3.58143E-05	3.73153E-05	0.00002662	3.08422E-05	3.22913E-05	2.98382E-05
chromosome_1_7553839_7554178	4.43669E-05	3.50538E-05	2.43641E-05	3.02375E-05	5.34825E-05	4.06885E-05
scaffold_25_9158_9426	4.11597E-05	8.81999E-05	3.56438E-05	0.000029028	5.04552E-05	0.000048148
chromosome_14_1262217_1262484	4.06251E-05	4.07076E-05	2.30105E-05	2.78185E-05	0.000052978	3.25508E-05
chromosome_4_599513_599553	0.00012241	4.18384E-05	5.63984E-05	0.000026609	4.18778E-05	4.47573E-05
chromosome_16_3391455_3391723	3.10034E-05	8.81999E-05	4.01556E-05	0.000026609	6.20599E-05	5.35732E-05
chromosome_4_210461_210966	1.76399E-05	1.75269E-05	1.26332E-05	0.000026609	1.76593E-05	0.000062389
chromosome_13_2954208_2954463	3.52797E-05	4.80576E-05	2.57177E-05	2.60042E-05	5.04552E-05	4.40792E-05
chromosome_12_4271406_4271681	3.10034E-05	3.10961E-05	1.84987E-05	2.47947E-05	4.89416E-05	3.59415E-05
chromosome_12_1932826_1933105	5.34541E-05	3.84461E-05	2.30105E-05	2.29805E-05	5.34825E-05	3.52634E-05
chromosome_3_5171964_5171988	7.48358E-06	7.34999E-06	4.51187E-06	0.000021771	4.59143E-05	2.23787E-05
chromosome_1_7531927_7532218	1.22944E-05	1.92231E-05	8.57255E-06	0.000016933	2.52276E-05	3.32289E-05
chromosome_13_2001068_2001202	4.16942E-05	0.000026573	2.12058E-05	1.26997E-05	1.11002E-05	1.28847E-05
chromosome_13_1865813_1866090	2.67271E-06	5.65384E-07	2.25594E-06	0.000012095	4.03642E-06	1.35628E-05
chromosome_17_4707939_4708088	1.06908E-06	1.69615E-06	4.51187E-07	1.14902E-05	7.56828E-06	1.62754E-05
chromosome_11_1535091_1535115	1.76399E-05	0.0000147	1.35356E-05	9.07124E-06	0.000010091	0.000018988
chromosome_4_471570_471639	9.62174E-06	2.26154E-06	3.6095E-06	7.25699E-06	4.03642E-06	1.28847E-05
chromosome_4_586361_586389	1.38981E-05	3.3923E-06	5.86543E-06	6.65224E-06	8.57739E-06	7.45956E-06
chromosome_12_1223802_1223826	2.13817E-06	5.65384E-07	9.02374E-07	0.000004838	4.03642E-06	3.39071E-06
chromosome_8_2488474_2488557	5.34541E-07	3.95769E-06	1.80475E-06	4.23325E-06	2.52276E-06	1.35628E-06
chromosome_4_3100623_3100751	4.27633E-05	0.000033923	3.33878E-05	3.6285E-06	1.0091E-06	8.1377E-06
chromosome_12_9189876_9190041	4.27633E-06	5.08845E-06	4.96306E-06	3.02375E-06	1.21093E-05	5.42513E-06
chromosome_13_4029649_4029686	5.34541E-07	0	0	3.02375E-06	2.01821E-06	4.74699E-06
chromosome_10_1462445_1462480	0	5.65384E-07	0	3.02375E-06	5.04552E-07	6.78141E-07
chromosome_11_2733167_2733188	0	0	4.51187E-07	3.02375E-06	4.03642E-06	8.1377E-06
chromosome_1_7070566_7070651	3.74179E-06	1.13077E-06	3.6095E-06	0.000002419	7.06373E-06	2.71257E-06
chromosome_5_700892_700912	1.60362E-06	5.65384E-07	0	0.000002419	1.51366E-06	4.06885E-06
chromosome_12_2578489_2578514	5.34541E-07	2.26154E-06	2.70712E-06	0.000002419	1.66502E-05	4.74699E-06
chromosome_17_1116273_1116293	5.34541E-07	0	4.51187E-07	0.000002419	1.0091E-06	5.42513E-06
chromosome_16_7263002_7263091	1.60362E-06	2.26154E-06	4.51187E-07	1.81425E-06	0	1.35628E-06
chromosome_6_3278336_3278363	0	0	0	1.81425E-06	2.01821E-06	1.35628E-06
chromosome_10_6166171_6166271	3.20725E-06	2.82692E-06	9.02374E-07	1.2095E-06	4.54097E-06	4.74699E-06
chromosome_2_7331851_7331871	1.06908E-06	5.65384E-07	0	1.2095E-06	5.04552E-06	0
chromosome_3_4155052_4155072	5.34541E-07	0	0	1.2095E-06	0	6.78141E-07
chromosome_8_4479431_4479453	5.34541E-07	0	0	1.2095E-06	0	0
chromosome_12_4529745_4529821	5.34541E-07	5.65384E-07	0	1.2095E-06	0	2.71257E-06
chromosome_7_3451388_3451654	1.60362E-06	1.13077E-06	0	6.04749E-07	0	6.78141E-07
chromosome_1_7911753_7911773	5.34541E-07	0	0	6.04749E-07	0	1.35628E-06
chromosome_7_32808_32831	0	0	0	6.04749E-07	0	0
chromosome_14_2636093_2636118	0	1.69615E-06	9.02374E-07	6.04749E-07	1.51366E-06	3.39071E-06
chromosome_7_4614400_4614433	3.20725E-06	0	4.51187E-07	0	3.02731E-06	2.71257E-06
chromosome_1_781444_781469	1.60362E-06	1.13077E-06	4.51187E-07	0	1.51366E-06	3.39071E-06
chromosome_10_4788545_4788612	1.06908E-06	5.65384E-07	0	0	2.52276E-06	6.78141E-07
chromosome_6_6841140_6841160	5.34541E-07	0	0	0	1.51366E-06	0
chromosome_6_3067367_3067455	0	0	0	0	0	0
chromosome_7_4386254_4386279	0	0	0	0	0	0
chromosome_7_4969004_4969026	0	0	4.51187E-07	0	0	0
chromosome_8_1168464_1168491	0	0	0	0	0	6.78141E-07
chromosome_11_3677466_3677486	0	0	0	0	0	0
chromosome_13_249543_249565	0	0	0	0	0	0
chromosome_14_1045259_1045280	0	0	0	0	5.04552E-07	6.78141E-07
chromosome_16_4708166_4708186	0	5.65384E-07	0	0	0	0
chromosome_16_5830783_5830805	0	0	0	0	0	0
chromosome_17_544133_544153	0	0	4.51187E-07	0	5.04552E-07	0

Supplementary table 2: Normalised miRNA abundance (total RNA)

	Parent-1	Parent-2	Parent-3	AGO3-25-1	AGO3-25-2	AGO3-25-3
chromosome_5_3227666_3227753_+	0.039868668	0.061289854	0.049587667	0.149174395	0.229784273	0.30839322
chromosome_10_3399870_3399999_	0.423775702	0.350977394	0.323608037	0.127655484	0.090263553	0.140683464
chromosome_12_6402226_6402307_	0.142005207	0.093417354	0.088144075	0.030168132	0.037247696	0.0275766
chromosome_6_6776108_6776193_+	0.116322702	0.091328506	0.062143571	0.013900142	0.033012295	0.024568244
chromosome_9_6365928_6366014_-	0.033072328	0.020796773	0.019119217	0.013319142	0.014704727	0.016900604
chromosome_17_6144120_6144204_	0.030564383	0.026299104	0.020880305	0.011060917	0.011276068	0.011006182
chromosome_16_185088_185174_-	0.077315542	0.037874379	0.032474133	0.007322788	0.004565433	0.006200149
chromosome_4_3694793_3694875_-	0.013200214	0.008263686	0.006286105	0.005218034	0.008269117	0.009159589
chromosome_5_1790702_1790791_+	0.013449094	0.010281207	0.007933048	0.00509745	0.00330948	0.002996127
chromosome_3_6573899_6573980_-	0.014320175	0.005206835	0.003212355	0.003814865	0.000476712	0.000599225
chromosome_2_2217872_2218043_-	0.008270475	0.005869153	0.005209885	0.003310602	0.003419491	0.003460833
chromosome_14_2347075_2347211_	0.011706934	0.012227402	0.008438546	0.002751527	0.005693039	0.003876622
chromosome_13_3152367_3152452_	0.003063139	0.003362536	0.001687709	0.002630942	0.004134558	0.004952782
chromosome_1_7070552_7070605_-	0.012338707	0.003494999	0.004957136	0.002323999	0.002365224	0.003473062
chromosome_13_2001067_2001197_	0.009735038	0.004401865	0.004932676	0.002181489	0.001356795	0.002445818
chromosome_13_2127530_2127632_	0.001876173	0.004136938	0.00181816	0.001545678	0.002841936	0.000819349
chromosome_14_3218783_3218866_	0.00252709	0.013429764	0.001573565	0.001479905	0.009259211	0.002213465
chromosome_1_5106349_5106475_+	0.010539112	0.007081704	0.005796914	0.001414131	0.000852581	0.001332971
chromosome_4_3100624_3100751_+	0.011706934	0.005390247	0.004484251	0.001315471	0.001164277	0.001919967
chromosome_8_121858_121942_-	0.00501589	0.003046661	0.002755776	0.001172962	0.00119178	0.00107616
chromosome_6_7063792_7063881_-	0.002077191	0.001314446	0.00110068	0.001085263	0.001274287	0.001687615
chromosome_7_4631481_4631544_-	0.000277597	0.000438149	0.000481038	0.000515226	0.000577555	0.000856036
chromosome_2_9129508_9129593_-	0.001876173	0.000957813	0.000945769	0.000460415	0.000476712	0.000513622
chromosome_2_9173306_9173389_+	0.010634836	0.005257783	0.005845833	0.00043849	0.001145942	0.000745975
chromosome_1_3724965_3725113_+	0.000114868	0.000132464	0.000195676	0.000416566	0.000265859	0.000611455
chromosome_7_5632709_5632783_-	0.002316499	0.001192172	0.000823472	0.00033983	0.000476712	0.000256811
chromosome_2_739310_739389_+	0.000526477	0.000427959	0.000407659	0.000317905	0.000403372	0.000635913
chromosome_7_2372822_2372907_-	0.0000765785	0.003107798	0.000244596	0.000306943	0.002970281	0.000207895
chromosome_10_6282484_6282565_	0.00049776	0.000142653	0.00020383	0.000153472	0.0000550052	0.0000978327
chromosome_4_457829_457914_-	0.000469043	0.00021398	0.000130451	0.000131547	0.000256691	0.000293498
chromosome_7_2371057_2371142_-	0.0000191446	0.001100466	0.0000978382	0.0000986603	0.000907586	0.000122291
chromosome_2_8349161_8349264_+	0.000315886	0.000142653	0.000220136	0.0000328868	0.000119178	0.0000611455
chromosome_7_5926395_5926482_+	0	0.000132464	0	0.0000109623	0.0000825078	0
chromosome_16_828236_828325_-	0.00012444	0.000101895	0.0000570723	0	0.0000183351	0.0000244582
chromosome_16_4056196_4056281_	0	0	0	0	0	0
chromosome_17_5149811_5150023_	0	0	0	0	0	0
chromosome_2_9101133_9101252_+	0	0	0	0	0	0
chromosome_4_3820425_3820510_+	0	0	0	0	0	0
chromosome_6_3067368_3067456_+	0	0	0	0	0	0
chromosome_7_4386252_4386309_-	0	0	0	0	0	0
chromosome_8_126807_126910_-	0	0	0	0	0	0
scaffold_24_82182_82317_+	0	0	0	0	0	0

Supplementary table 3: Normalised miRNA abundance (AGO-IP)

IP-Antibody	α2/3-PAZ					α-At-AGO1	
	Parent-1	Parent-2	Parent-3	ago3-25-1	ago3-25-2	Parent-1	ago3-25-2
chromosome_5_3227666_3227753_+	0.335874934	0.090488075	0.983544607	0	0	0	0.000044817
chromosome_3_6573899_6573980_-	0.000491548	0	0	0	0	0	0
chromosome_13_3152367_3152452_	0.000196619	0	0.000574025	0.000305099	0.0000885772	0.002945416	0.001523779
chromosome_13_2001067_2001197_	0.0000491548	0	0.000287013	0.000381374	0.0000885772	0.001453582	0.000313719
chromosome_1_3724965_3725113_+	0	0	0	0	0	0	0
chromosome_1_5106349_5106475_+	0	0	0	0	0.0000885772	0.0000382522	0.000044817
chromosome_1_7070552_7070605_-	0	0	0	0	0	0	0
chromosome_10_3399870_3399999_	0	0	0.0000478354	0	0	0	0.000044817
chromosome_10_6282484_6282565_	0	0	0	0	0	0	0
chromosome_12_6402226_6402307_	0	0	0	0	0	0.000344269	0.000179268
chromosome_13_2127530_2127632_	0	0	0	0	0	0	0
chromosome_14_2347075_2347211_	0	0	0.008993064	0	0	0.000153009	0.000044817
chromosome_14_3218783_3218866_	0	0	0.006314279	0	0	0	0.0000896341
chromosome_16_185088_185174_-	0	0.081766913	0	0	0.0000885772	0	0
chromosome_16_828236_828325_-	0	0	0	0	0	0	0
chromosome_16_4056196_4056281_	0	0	0	0	0	0	0
chromosome_17_5149811_5150023_	0	0	0	0	0	0	0
chromosome_17_6144120_6144204_	0	0	0	0	0	0.0000382522	0.000044817
chromosome_2_739310_739389_+	0	0	0	0	0	0	0
chromosome_2_2217872_2218043_-	0	0	0	0	0	0.0000382522	0
chromosome_2_8349161_8349264_+	0	0	0	0	0	0	0
chromosome_2_9101133_9101252_+	0	0	0	0	0	0	0
chromosome_2_9129508_9129593_-	0	0	0	0	0	0	0
chromosome_2_9173306_9173389_+	0	0	0	0	0	0	0
chromosome_4_457829_457914_-	0	0	0	0	0	0	0
chromosome_4_3100624_3100751_+	0	0	0.0000478354	0	0	0.0000382522	0
chromosome_4_3694793_3694875_-	0	0	0	0	0	0	0
chromosome_4_3820425_3820510_+	0	0	0	0	0	0	0
chromosome_5_1790702_1790791_+	0	0	0.0000478354	0	0	0.0000382522	0
chromosome_6_6776108_6776193_+	0	0	0.0000956709	0.0000762747	0	0.000267765	0.000044817
chromosome_6_7063792_7063881_-	0	0	0	0	0	0	0
chromosome_6_3067368_3067456_+	0	0	0	0	0	0	0
chromosome_7_2371057_2371142_-	0	0	0	0	0	0	0
chromosome_7_2372822_2372907_-	0	0	0	0	0	0	0
chromosome_7_4386252_4386309_-	0	0	0	0	0	0	0
chromosome_7_4631481_4631544_-	0	0	0	0	0	0	0
chromosome_7_5632709_5632783_-	0	0	0	0	0	0	0
chromosome_7_5926395_5926482_+	0	0	0	0	0	0	0
chromosome_8_121858_121942_-	0	0	0	0	0	0	0
chromosome_8_126807_126910_-	0	0	0	0	0	0	0
chromosome_9_6365928_6366014_-	0	0	0.0000478354	0.0000762747	0	0.0000765043	0.000044817
scaffold_24_82182_82317_+	0	0	0	0	0	0	0
